# Modelling daisy quorum drive: A short-term bridge across engineered fitness valleys

**DOI:** 10.1371/journal.pgen.1011262

**Published:** 2024-05-16

**Authors:** Frederik J. H. de Haas, Léna Kläy, Florence Débarre, Sarah P. Otto

**Affiliations:** 1 Biodiversity Research Center, Department of Zoology, University of British Columbia, Vancouver BC, Canada; 2 Institute of Ecology and Environmental Sciences Paris (IEES Paris), Sorbonne Université, CNRS, IRD, INRAE, Université Paris Est Creteil, Université de Paris, Paris Cedex 5, France; Peking University, CHINA

## Abstract

Engineered gene-drive techniques for population modification and/or suppression have the potential for tackling complex challenges, including reducing the spread of diseases and invasive species. Gene-drive systems with low threshold frequencies for invasion, such as homing-based gene drive, require initially few transgenic individuals to spread and are therefore easy to introduce. The self-propelled behavior of such drives presents a double-edged sword, however, as the low threshold can allow transgenic elements to expand beyond a target population. By contrast, systems where a high threshold frequency must be reached before alleles can spread—above a fitness valley—are less susceptible to spillover but require introduction at a high frequency. We model a proposed drive system, called “daisy quorum drive,” that transitions over time from a low-threshold daisy-chain system (involving homing-based gene drive such as CRISPR-Cas9) to a high-threshold fitness-valley system (requiring a high frequency—a “quorum”—to spread). The daisy-chain construct temporarily lowers the high thresholds required for spread of the fitness-valley construct, facilitating use in a wide variety of species that are challenging to breed and release in large numbers. Because elements in the daisy chain only drive subsequent elements in the chain and not themselves and also carry deleterious alleles (“drive load”), the daisy chain is expected to exhaust itself, removing all CRISPR elements and leaving only the high-threshold fitness-valley construct, whose spread is more spatially restricted. Developing and analyzing both discrete patch and continuous space models, we explore how various attributes of daisy quorum drive affect the chance of modifying local population characteristics and the risk that transgenic elements expand beyond a target area. We also briefly explore daisy quorum drive when population suppression is the goal. We find that daisy quorum drive can provide a promising bridge between gene-drive and fitness-valley constructs, allowing spread from a low frequency in the short term and better containment in the long term, without requiring repeated introductions or persistence of CRISPR elements.

## Author summary

Gene drive based on CRISPR-Cas9 technology holds great promise for addressing many pressing environmental and humanitarian problems, ranging from reducing the incidence of disease to protecting species at risk. These potential benefits come with substantial concerns about engineered elements having unintended consequences and expanding beyond targeted populations. We model an alternative, called daisy quorum drive, where CRISPR-Cas9 elements are only present temporarily, as part of a daisy chain that drives a second construct to high frequency but that is counterselected and exhausts over time. This second construct carries out an intended genetic modification but is unfit at low frequencies. Thus, once drive has exhausted, a fitness valley is created where individuals carrying only wildtype alleles or the second construct have high fitness, but crosses between them do not. We show that this “fitness-valley construct’’, once established by drive, can persist and modify local populations, but its expansion to other populations is inhibited because the fitness-valley construct is unfit at low frequency. This design can be used when it is challenging to breed a species in the large numbers needed to establish a fitness-valley construct, while avoiding the long-term presence of gene drive elements.

## 1 Introduction

The introduction of gene drive systems into sexually reproducing species can spread desirable gene modifications into a population and potentially solve many pressing humanitarian and environmental problems, ranging from public health and agriculture to conservation [1]. Gene drive occurs when alleles bias meiotic transmission in their favour. Gene drive occurs naturally, but synthetic gene drives can now be engineered and potentially released for population control [2]. Example applications include modifying the genome of disease-bearing mosquito species to reduce their capacity to serve as a vector (e.g., reducing the number of female mosquitoes, their longevity, or their ability to support development and transmission of the pathogen) [3]. Other examples include driving genes that protect species at risk by eradicating invasive species or reducing pest damage in agriculture by replacing resistant alleles with their ancestral equivalents to restore vulnerability and reduce application of pesticides or herbicides [1].

Because their segregation advantage allows gene drive systems to increase when rare, however, they pose a risk of spreading outside of the species and region of interest. As summarised below, numerous countermeasures have been devised that limit where and when drive can occur, including daisy chains [4]. In a daisy chain, a series of loci each drive the next element in the chain using CRISPR-Cas9 technology. Because the first element is not driven and all drive elements reduce fitness, eventually drive exhausts. This limits the risk posed by gene drive systems but can also require repeated introductions to achieve the goals of the release program.

An alternative approach to gene drive takes advantage of natural or engineered constructs that generate a “fitness valley,” where individuals have low fitness when carrying a combination of the construct and wildtype alleles but high fitness with either allele type alone (as detailed below). Unlike gene drive constructs that spread when rare (low threshold systems), fitness-valley constructs have a high threshold, where the constructs must be introduced at high enough frequencies before they can establish. For example, infection by *Wolbachia* bacteria can cause male sterility in mosquitoes when infected males mate with uninfected females. This incompatibility creates a fitness valley, such that *Wolbachia* spreads only when introduced above a threshold frequency. Because infection alters the ability of mosquitoes to spread disease, the introduction in high numbers of *Wolbachia*-infected mosquitoes has already been used to control dengue, Zika, and other arboviruses in the wild [5].

**Fig 1:**
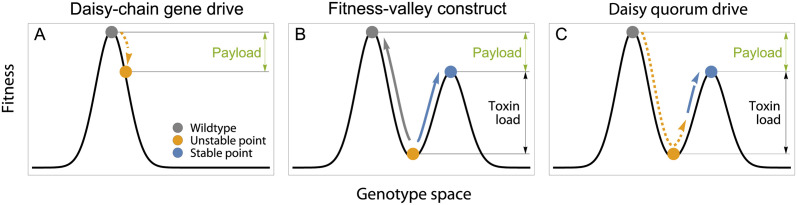
Conceptual overview of the different control systems. (A) Daisy-chain drive temporarily introgresses a cargo that carries a deleterious element that reduces fitness (the “payload”), ultimately reverting back to the wildtype state (grey). (B) The dynamics with a fitness valley is frequency dependent: left of the valley the population is driven back to a wildtype composition, while right of the valley it evolves toward the lower peak expressing the payload. (C) The proposed design (daisy quorum drive) includes both systems and uses the temporary daisy chain to drive a fitness-valley construct past the threshold frequency, after which it evolves toward the other peak with the desired payload.

The goal of this paper is to model “daisy quorum drive” (Fig 1), a combined approach that links a self-exhausting homing drive system known as a daisy chain [4] with a fitness-valley construct, here a two-locus toxin-antidote system [6], that without drive requires both antidote alleles to be at a high enough frequency to spread (requiring a “quorum”). The concept of daisy quorum drive was first proposed by Min et al. [7] as a promising approach to reduce the risks of gene drive while retaining its advantages. In brief, daisy quorum drive transitions over time from a low to a high threshold system, which has a lower chance of expanding to non-target populations than with homing gene drive [8, 9]. Nevertheless, the initial size of the release needed to alter a population is lower than with other fitness-valley constructs, which allows the daisy quorum drive design to be used even in species where breeding and releasing large numbers of individuals is challenging. Furthermore, by linking the drive loci (e.g., CRISPR-Cas9) to deleterious alleles, selection is predicted to eliminate these drive elements over time.

We next provide more detailed information about both gene drive and fitness-valley systems before discussing how these elements are combined in daisy quorum drive.

*Homing gene drive—* Biased transmission can now be genetically engineered using a variety of constructs, including CRISPR-Cas9 homing drives that cut target sites and allow homology-directed repair (HDR) to copy the driver, thereby increasing in frequency. Introduced at low frequency in a natural population, homing gene drive systems can spread rapidly even when selected against, thanks to their “super-Mendelian inheritance” [10–14].

To engineer drive using CRISPR, a construct is built that includes an endonuclease (often Cas9) and a guide RNA (gRNA), which are transcribed in the germline. The endonuclease triggers a double-strand break at the complementary site of the gRNA, which can be repaired by either one of two pathways: homologous recombination via HDR or non-homologous end joining (NHEJ). When the double-strand break is repaired by homologous recombination, the result is that the sequence homologous to the target of the double-strand break is copied. When the homologous chromosome carries the CRISPR-Cas9 cassette itself, the result is a conversion of heterozygous cells into homozygotes carrying the cassette. If cleavage with homologous repair occurs at rate *δ*, the result is a form of meiotic drive with a greater fraction of gametes carrying the construct than expected under Mendelian inheritance (modeled as heterozygotes producing a fraction $\frac{1}{2}\left(1+\delta \right)$ of gametes carrying the driven allele). High conversion rates have been achieved in the lab by the CRISPR-Cas9 system for yeast (*δ* > 0.99, [15]), fruit flies (*δ* > 0.85, [16]), and the malaria vector mosquito, *Anopheles stephensi* (*δ* > 0.90, [17]), although the strength of drive can depend strongly on the genetic background [18].

In NHEJ, the double-strand break ends are directly ligated without the use of the homologous template. NHEJ often introduces mutations, generating sequences that may no longer be recognized by the gRNA and hence are resistant to future drive, limiting the potential of CRISPR-Cas9 based systems [4, 19].

Two general applications of engineered homing gene drive have been proposed: population modification and population suppression. The goal of population modification is to change the composition of a target population. For example, *Anopheles stephensi* mosquitoes expressing m1C3, m4B7, or m2A10 single-chain antibodies (scFvs) have significantly lower levels of infection compared to controls when challenged with *Plasmodium falciparum*, a human malaria pathogen [3]. Introduction of scFvs genes could thus be used to modify mosquito populations in the quest to control malaria. By altering the genome of a species rather than eliminating it, population modification potentially causes less disruption to ecological networks than population suppression, discussed next.

The goal of population suppression is to use gene-drive systems to purposefully reduce the size of the target population by lowering fertility or survival. In the laboratory, population suppression was successfully shown in *Anopheles gambiae* [12]. This study used a CRISPR-Cas9 gene drive targeting a doublesex gene (Agdsx) that leads to a biased reproductive sex ratio by rendering homozygous females at Agdsx completely sterile. Within 7–11 generations, egg production was reduced to the point of total population collapse. One concern about population suppression is that it may have unintended impacts on an ecosystem. The ecological role of mosquitoes, for example, is poorly understood in most ecosystems, but they are known to function as pollinators, are important components of many aquatic food webs, and transfer substantial amounts of energy between aquatic and terrestrial food webs as they emerge [20, 21]. Relative to chemical control, however, suppression drives could reduce these ecological impacts by targeting the few mosquito species that are important disease vectors.

Under a wide range of parameter conditions, homing drive systems require a low initial number of transgenic individuals to spread due to meiotic drive, but as a consequence they are also highly invasive [10, 22–25].

Spatial confinement is a highly desirable, if not essential, attribute for use of gene drive systems outside of laboratory settings [1, 26]. Designing constructs that limit the extent of drive over time and spread through space reduces the risk of accidental release [27, 28] and spread from field trials [29].

Numerous countermeasures have been proposed to remove the homing drive systems after their initial spread: i) temporary drives relying on the segregation of the construct at independent loci in the genome, such as split drives [15], daisyfields [30], or daisy chains [4] or ii) drive reversal approaches relying on the introduction of another drive construct at a later stage, such as CHACRs [31], ERACRs [31], CATCHAs [32], or of a phage-derived protein (AcrIIA4) that inactivates CRISPR [33]. These countermeasures also generally eliminate the benefits for population modification or suppression. In section 2.6, however, we discuss other designs that aim to maintain spatially confined constructs, comparing these designs to daisy quorum drive.

**Fig 2:**
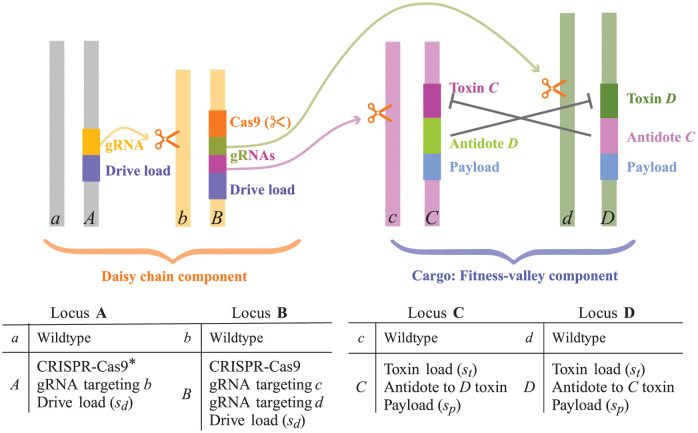
Four-locus daisy quorum drive. The daisy-chain component consists of loci **A** and **B**, with engineered homing gene drive alleles (*A* and *B*) that carry deleterious elements reducing fitness (drive load of *s*_*d*_). The fitness-valley component consists of two loci **C** and **D**, with alleles *C* and *D* engineered to reduce fitness by expressing a toxin (toxin load of *s*_*t*_) and an element modifying the population (the payload, which reduces fitness by *s*_*p*_). Because the toxin is only expressed if the antidote, carried by the other fitness-valley locus, is absent, alleles *C* and *D* create a fitness valley when they are both rare, with fitness interactions given by Table 1. The gRNA of locus **A** is complementary to the wildtype allele *b*, and locus **B** carries two gRNAs to cut the wildtype alleles *c* and *d* at the fitness-valley loci. Longer daisy chains of size *n* can be designed by repeating locus **A** for **A_1_**,…, **A_*n*−1_**, each carrying a gRNA targeting the next locus in the chain. *To reduce the rate of resistance mutations, we illustrate a case where the first locus in the chain does not carry CRISPR-Cas9, whose functionality is provided only when the construct is present at the target locus; one could also design the gRNAs for each locus to be in the construct for that locus itself, preventing cutting when the construct is not present [34].

While all of these countermeasures could potentially limit gene drive in space and time, we model daisy quorum drive (as illustrated in Fig 2, as described below) using a daisy chain component [4], with multiple interdependent homing-based drive constructs across the genome, each bearing a cost (drive load). Every locus in the daisy chain drives the next locus in the chain (i.e., the gRNA of one locus recognizes and guides Cas9 to cut the wildtype allele at the next locus in the chain). The last element in the chain recognizes and cuts the wildtype alleles at the cargo loci, which in our model is a two-locus fitness-valley construct (Fig 2). The more loci in the daisy chain, the longer the drive will last and the higher up in frequency it will push the cargo (S1 Appendix, section S1.1; [4]). Because no element in the daisy chain can drive itself and the first element is not driven, all CRISPR-Cas9 elements are ultimately expected to be lost from the population due to natural selection against the drive load, exhausting the drive.

*Fitness valleys—* Unlike homing gene drive, fitness-valley constructs decline in frequency when rare but can rise in frequency once sufficiently common, either by introducing the construct at high frequency (as in the *Wolbachia* system; [5]) or by driving it to high frequency using a daisy chain (as in our model of daisy quorum drive).

Fitness valleys were first described for reciprocal chromosomal translocation (RCL) systems [35, 36], with translocation heterozygotes having lower fitness than either homozygote (underdominance). Various toxin-antidote and related systems have since been proposed to generate a fitness valley, including killer-rescue systems [37], one-locus one-toxin-antidote (1L1T) systems [38], one-locus two-toxin-antidote (1L2T) systems [6, 39], and two-locus two-toxin-antidote (2L2T) systems [6, 39]. If introduced above their frequency threshold, these systems are predicted to spread [6, 35, 36, 38]. As a much higher initial frequency is needed with one-locus than two-locus constructs [6, 9], we focus here on two-locus constructs, but both in principle could be used with daisy quorum drive. Although previously called “underdominant” constructs, we use “fitness-valley” constructs to include cases where epistasis between loci, not dominance within, generates the fitness valley.

Because fitness-valley constructs are at a disadvantage when rare, they are less likely to expand to other locations if introduced by the occasional migrant. Two-patch models exploring a variety of one- and two-locus constructs have shown that fitness-valley constructs remain largely confined to the patch in which they are initially introduced [8, 40–43], as long as migration is sufficiently infrequent. Spatial confinement has also been demonstrated in continuous but heterogeneous environments, where regions of low dispersal can restrict spread [9, 42, 44, 45]. Maintenance of a fitness-valley construct in a confined region of space is generally not possible, however, in continuous and homogenous environments [9, 44, 45], essentially because of the high rate of gene flow between neighboring individuals, and the construct is expected to either contract or expand indefinitely in space.

*Daisy quorum drive—* Daisy quorum drive uses a self-exhausting daisy chain to drive a fitness-valley construct to high enough frequency that it can spread even without drive (Fig 1). Technically, gene drive temporarily eliminates the fitness valley because of the segregation advantage of the construct, but the valley emerges when drive has exhausted.

Once homing gene drive is over, the above attributes of fitness-valley constructs apply equally well to daisy quorum drive systems. In particular, in the long term, we expect the fitness-valley component of daisy quorum drive to remain spatially restricted, as long as migration rates are sufficiently low. While gene drive is ongoing, however, the dynamics of daisy quorum drive are unique. The threshold for the construct to rise in frequency is no longer a constant but changes over time, rising as the drive exhausts. Thus, to be effective, we must determine the conditions for the cargo to have reached a sufficient frequency before homing drive is over. Daisy quorum drive can also be used even when fitness-valley constructs are expected to contract over time by using the driving phase to establish the construct in space. Following this phase, the spatial range is expected to contract over time, either becoming stabilized if dispersal is heterogeneous enough or disappearing if not.

Here we use mathematical models and spatial simulations to evaluate the risks and potential benefits of daisy quorum drive and to guide design choices in light of different objectives and concerns. Models are an important first step in the evaluation process for gene drive, allowing exploration of the features that allow spread to occur as intended but not beyond.

Specifically, we seek to clarify when daisy quorum drive will transiently drive a fitness-valley construct to a high enough frequency that it becomes established, while ultimately leaving the population without any homing-based gene-drive elements. We also determine the spatial dynamics of daisy quorum drive, considering various ways in which populations may be arrayed over space: discrete or continuous space, with homogeneous or heterogeneous dispersal rates. We find that the daisy quorum design can allow persistent modification of a target population(s), while inhibiting spread to non-target populations. Advantages of daisy quorum drive include: i) the small initial release needed, ii) the transition to a high threshold frequency that improves spatial confinement, and iii) the eventual elimination of the CRISPR-Cas9 drive system. The transient nature of the daisy chain also limits the time frame over which alleles resistant to drive can accumulate, relative to standard homing gene drive [4].

Importantly, the simulations consider only the best case scenario, where the genetic elements work as intended, without off-target effects, compensatory mutations, or unexpected genotype-by-environment interactions. The models explored here thus provide insight into the design features of daisy quorum drive that lower, but do not eliminate, the risks involved. All gene drive systems, including daisy quorum drive, come with associated risks that must be evaluated and mitigated prior to use, with meaningful engagement, collaboration, and consent of local communities and governments [1, 26, 46].

## 2 Methods and results

We formulate a model that considers the evolution of a large population of diploid organisms, assuming random mating and discrete-time dynamics. We first introduce a deterministic model for a single population and then consider spatially distributed populations with migration and with stochasticity. We refer to the loci in our model by bold letters (e.g., **A**) and to the alleles by italic letters (e.g., *A* or *a*).

### 2.1 Drive design

We model daisy quorum drive with two components (Fig 2). The first daisy-chain component uses self-limiting CRISPR technology to temporarily drive the second fitness-valley component past its threshold for spread without drive [7]. All CRISPR-Cas9 components include elements that reduce fitness (drive load), so that they are counterselected once the daisy chain exhausts, with the goal of leaving only the second fitness-valley component within the genome. Fig 2 illustrates a daisy-chain with two loci (**A** and **B**), but we also consider longer chains, which drive for longer periods and can push cargoes to higher frequencies ([4]; S1 Appendix, section S1.1).

The transgenic alleles of the daisy chain consist of a CRISPR-Cas9 complex, one or more gRNAs, and an element that reduces fitness by *s*_*d*_ (the drive load). Here we assume multiplicative fitness effects across alleles and loci, although other designs are possible as long as each driver allele is selectively disfavoured in the absence of drive. The drive load ensures that all CRISPR-Cas9 elements are counterselected and eventually disappear from the system. Until they disappear, the gRNA at locus *i* in the chain guides the Cas9 nuclease to the wildtype allele at locus *i*+ 1, inducing a double-strand break that is repaired by homologous recombination with probability *δ* (see [34] for alternative designs). We assume that homology-dependent repair is efficient in the focal species and do not incorporate resistance to Cas9 nuclease because of the transient nature of the daisy chain, which limits the time frame over which resistant alleles can rise in frequency [4]. As noted by [4], some taxa have very inefficient homology-dependent repair (high rates of NHEJ); in such cases, cleavage can rapidly generate resistant alleles and impede the daisy chain, hampering its use for daisy quorum drive.

The final element in the chain drives the “cargo,” which in our model is the second component of daisy quorum drive, a two-locus toxin-antidote system that generates a fitness-valley (loci **C** and **D**). The two-locus fitness-valley component is structured following the design proposed by [6]. Two transgenic alleles each consist of three elements: a toxin, an antidote, and a payload. The two engineered alleles are carried on non-homologous chromosomes, each of which produces a toxin unless the antidote on the other transgenic allele is also present, which suppresses the expression of the toxin (e.g., by blocking transcription). In the absence of the matching antidote, the toxin is expressed, reducing fitness by *s*_*t*_ (Table 1). Consequently, when rare, alleles *C* and *D* decline in frequency because their toxins are rarely suppressed. At high frequency, however, both engineered alleles spread because the wildtype alleles *c* and *d* fail to carry antidotes to the now-common toxins. Once at high frequency, the payload modifies the population (e.g., changing the life cycle or habitat preferences of the species), reducing fitness by *s*_*p*_. This scheme creates a multi-peaked fitness regime, promoting the state of carrying both engineered alleles (*CD*) or neither (*cd*) and selecting against individuals carrying only one transgenic allele. A version of this two-locus fitness-valley construct was analysed by Davis et al. [6] for the special case of a lethal toxin load (*s*_*t*_ = 1) without a payload (*s*_*p*_ = 0). Min et al. [7] proposed a slightly different daisy quorum drive system, generating a fitness-valley construct by swapping haploinsufficient alleles at two loci (see section 2.6).

**Table 1:** Relative fitness for the two-locus fitness-valley component. The rows and columns denote the haplotypes (set of alleles inherited from each parent) of a diploid genotype. The toxin *s*_*t*_ is expressed in individuals carrying either a *C* or *D* allele but not both. The payload *s*_*p*_ is expressed if the individual carries at least one engineered allele (dominant payload). The resulting fitness landscape has two stable equilibria (*cd* or *CD* fixed), separated by a fitness valley caused by the toxin load.

	*cd*	*cD*	*Cd*	*CD*
*cd*	1	(1 − *s*_*t*_)(1 − *s*_*p*_)	(1 − *s*_*t*_)(1 − *s*_*p*_)	(1 − *s*_*p*_)
*cD*	(1 − *s*_*t*_)(1 − *s*_*p*_)	(1 − *s*_*t*_)(1 − *s*_*p*_)	(1 − *s*_*p*_)	(1 − *s*_*p*_)
*Cd*	(1 − *s*_*t*_)(1 − *s*_*p*_)	(1 − *s*_*p*_)	(1 − *s*_*t*_)(1 − *s*_*p*_)	(1 − *s*_*p*_)
*CD*	(1 − *s*_*p*_)	(1 − *s*_*p*_)	(1 − *s*_*p*_)	(1 − *s*_*p*_)

Unless otherwise stated, we assume that the loci involved are freely recombining and that the *ABCD* constructs are introduced into a focal population at a frequency of *f*_0_ immediately before reproduction, with remaining individuals carrying the wildtype alleles *abcd*.

As noted in the introduction, there has been extensive previous work on the spatial expansion of fitness-valley constructs in the absence of gene drive, starting with the seminal study by Barton of one-locus underdominance [44]. Examining a system similar to the two-locus fitness-valley constructs considered here, Dhole et al. demonstrated that the constructs could remain spatially localized and not spread to a neighboring patch in a two-patch model [8]. In a spatially continuous population, Champer et al. found that fitness-valley constructs, as designed, often contract when released in a spatially continuous population with homogenous dispersal (including RCL, 1L1T, 1L2T, and 2L2T systems); only the two-locus two-toxin-antidote (2L2T) system could persist and spread after a central release surrounded by wild-type individuals and only for a low enough payload [9]. Another two-locus fitness-valley system was recently developed and modeled, using engineered constructs bearing two genes isolated from *Wolbachia*, which causes male sterility when the male expresses one construct but the female does not express the antidote [47]. Modelling demonstrated that this *Wolbachia*-based construct could be confined in a patchy environment but would either spread or contract in a spatially continuous environment [47]. Once gene drive is exhausted, we expect the two-locus fitness valley component modeled here to exhibit similar behaviour to these other two-locus systems.

Fitness components resulting from the drive load, the toxin, and the payload are assumed to multiply together across loci to determine individual fitness. Table 1 illustrates the effect of loci **C** and **D** on individual fitness resulting from the toxin and payload, when the payload reduces fitness by *s*_*p*_ and is dominantly expressed. In S1 Appendix, we also explore different payload fitness functions, including multiplicative (section S1.5) and recessive (section S1.6) expression. To derive the relative fitness for each full genotype, including the drive load, the fitness of the **CD** genotype (Table 1) is multiplied by (1 − *s*_*d*_)^*j*^ where *j* equals the number of daisy chain alleles carried by the individual.

### 2.2 Dynamics in an isolated population

The recursion equations for the full four-locus daisy quorum system with 16 (= 2^4^) gamete types are too elaborate so we report them in an associated *Mathematica* file archived on Zenodo doi:10.5281/zenodo.10904198 (also available for download as a PDF). Here, we describe the equations for the daisy-chain component (**A** and **B**) and the fitness-valley components (**C** and **D**) in isolation (Fig 2). We use recursion equations to track the population through diploid selection followed by meiosis with drive and recombination.

Assuming random mating in a single isolated population, the recursion equations for the daisy chain, describing the frequency *X*_*ij*_ of the gamete *ij* at loci **A** and **B** with recombination rate *R* between them, are:

$$\begin{array}{ll} X_{ab}^{\prime } & =\frac{1}{\bar{W}}(X_{ab}^{2}+X_{aB}X_{Ab}{\left(1-s_{d}\right)}^{2}(1-\delta )R+X_{ab}X_{aB}(1-s_{d})+X_{ab}X_{Ab}(1-s_{d})\\ & \;\;\;\;\;+X_{ab}X_{AB}{\left(1-s_{d}\right)}^{2}(1-\delta )(1-R))\\ X_{aB}^{\prime } & \frac{1}{\bar{W}}(X_{aB}^{2}{\left(1-s_{d}\right)}^{2}+X_{aB}X_{Ab}{\left(1-s_{d}\right)}^{2}(1-R(1-\delta ))+X_{aB}X_{AB}{\left(1-s_{d}\right)}^{3}\\ & \;\;\;\;\;+X_{ab}X_{aB}(1-s_{d})+X_{ab}X_{AB}{\left(1-s_{d}\right)}^{2}(1-R(1-\delta )))\\ X_{Ab}^{\prime } & \frac{1}{\bar{W}}(X_{Ab}^{2}{\left(1-s_{d}\right)}^{2}+X_{aB}X_{Ab}{\left(1-s_{d}\right)}^{2}(1-\delta )(1-R)+X_{Ab}X_{AB}{\left(1-s_{d}\right)}^{3}(1-\delta )\\ & \;\;\;\;\;+X_{ab}X_{Ab}(1-s_{d})+X_{ab}X_{AB}{\left(1-s_{d}\right)}^{2}(1-\delta )R)\\ X_{AB}^{\prime } & \frac{1}{\bar{W}}(X_{AB}X_{ab}{\left(1-s_{d}\right)}^{2}(1-R(1-\delta ))+X_{AB}X_{Ab}{\left(1-s_{d}\right)}^{3}(1-\delta )+X_{AB}^{2}{\left(1-s_{d}\right)}^{4}\\ & \;\;\;\;\;+X_{aB}X_{Ab}{\left(1-s_{d}\right)}^{2}(R+\delta (1-R))+X_{aB}X_{AB}{\left(1-s_{d}\right)}^{3}), \end{array}$$ (1)

where *X*_*ab*_ + *X*_*aB*_ + *X*_*Ab*_ + *X*_*AB*_ = 1 and $\bar{W}$ is the average fitness (the sum of the numerators). These recursions follow from the mating and gamete production S1 Table and are equivalent to equations analysed by Noble et al. [4] except in discrete time. An automated algorithm for deriving the dynamical equations for longer daisy chains is provided in the *Mathematica* file on Zenodo (doi:10.5281/zenodo.10904198).

The recursion equations for the fitness-valley component, describing the frequency *X*_*ij*_ of the gamete *ij* at loci **C** and **D** with recombination rate *r* between them, are:

$$\begin{array}{l} X_{cd}^{\prime }=\frac{X_{cd}^{2}W_{cd,cd}+X_{cd}X_{cD}W_{cd,cD}+X_{cd}X_{Cd}W_{cd,Cd}+X_{cd}X_{CD}W_{cd,CD}-r\;dis}{\bar{W}}\\ X_{cD}^{\prime }=\frac{X_{cD}^{2}W_{cD,cD}+X_{cd}X_{cD}W_{cd,cD}+X_{cD}X_{CD}W_{cD,CD}+X_{cD}X_{Cd}W_{cD,Cd}+r\;dis}{\bar{W}}\\ X_{Cd}^{\prime }=\frac{X_{Cd}^{2}W_{Cd,Cd}+X_{cd}X_{Cd}W_{cd,Cd}+X_{Cd}X_{CD}W_{Cd,CD}+X_{cD}X_{Cd}W_{cD,Cd}+r\;dis}{\bar{W}}\\ X_{CD}^{\prime }=\frac{X_{CD}^{2}W_{CD,CD}+X_{cD}X_{CD}W_{cD,CD}+X_{Cd}X_{CD}W_{Cd,CD}+X_{cd}X_{CD}W_{cd,CD}-r\;dis}{\bar{W}}, \end{array}$$ (2)

where *X*_*cd*_ + *X*_*cD*_ + *X*_*Cd*_ + *X*_*CD*_ = 1, *W*_*genotype*_ represents the fitness of individuals of the given genotype as described in Table 1, $\bar{W}$ is the average fitness, and *dis* = *X*_*cd*_*X*_*CD*_*W*_*cd*,*CD*_ − *X*_*cD*_*X*_*Cd*_*W*_*cD*,*Cd*_ is the disequilibrium weighted by the fitness (see detailed outcome of each mating in S2 Table).

Assuming that the construct is introduced in individuals bearing the *CD* haplotype, the *C* and *D* alleles are initially equally frequent (*X*_*cD*_ = *X*_*Cd*_ at *t* = 0) and remain so over time according to Eq 2 ($X_{cD}^{\prime }=X_{Cd}^{\prime }$). Loss of the construct (*X*_*cd*_ = 1) and its fixation (*X*_*CD*_ = 1) both represent equilibria in the fitness-valley component of the dynamical equations 2, and when the payload is dominant, a local stability analysis indicates that both fixation states are locally stable. A third polymorphic equilibrium represents the co-existence of alleles *C* and *c*, as well as *D* and *d*, and is locally unstable. In S1 Appendix, section S1.3, we describe the location of the stable equilibria and the separatrix (the boundary separating two basins of attraction in a dynamical model) for different dominant payloads, *s*_*p*_, which determines the relative height of the fitness peaks, and different toxin loads, *s*_*t*_, which determines the valley depth (S2 Fig).

When the payload is not dominant, fixation of the construct is no longer a stable equilibrium; instead, alleles *C* and *D* rise when started above the separatrix but they do not fix (see S4 and S5 Figs for multiplicative and recessive payloads, respectively). Similarly, fixation of the two-locus fitness-valley construct studied by Dhole et al. [8] and Champer et al. [9] is unstable in a model with a multiplicative payload carried by only one of the transgenic alleles, *C* (given in S1 Appendix, section S1.8). Relative to these other fitness regimes, a dominant payload carried by both alleles *C* and *D* has the feature that it is expected to fix; thus, if technically feasible to engineer, it would be more robust to resistance evolving via reduced expression of the payload in heterozygous individuals carrying both transgenic and wildtype alleles. We focus on this configuration in the main text.

Although the unstable equilibrium on the separatrix is unwieldy, we can use it to determine when the threshold for spread of the fitness-valley construct is at the midpoint (${\hat{p}}_{C}={\hat{p}}_{c}={\hat{p}}_{D}={\hat{p}}_{d}=1/2$). For a dominant payload, a midpoint threshold is found when:

$$\begin{array}{c} s_{t}=\sqrt{\frac{rs_{p}^{2}}{2\left(1-s_{p}\right)\left(2r\left(1-s_{p}\right)-s_{p}\right)}}. \end{array}$$ (3)

With a more severe payload (larger *s*_*p*_), the unstable equilibrium rises, generating a higher-threshold construct with a smaller basin of attraction to fixation of the engineered fitness-valley construct, *X*_*CD*_ = 1 (i.e., $d{\hat{p}}_{i}/ds_{p}{|}_{{\hat{p}}_{i}=1/2}>0$ for *i* = {*C*, *D*}, causing the separatrix in S2 Fig to move right of centre). Higher payloads thus make it harder to drive such constructs into a population, but they also provide stronger fitness effects when the construct is able to fix. By contrast, with a stronger toxin load (larger *s*_*t*_), the unstable equilibrium falls, generating a lower threshold construct with a larger basin of attraction to fixation of *CD* (i.e., $d{\hat{p}}_{i}/ds_{t}{|}_{{\hat{p}}_{i}=1/2}<0$ for *i* = {*C*, *D*}, causing the separatrix in S2 Fig to move left of centre). While somewhat counterintuitive, this occurs because the toxin load can be suppressed by even one copy of the antidote allele, making haplotypes *Cd* and *cD* strongly selected against when *cd* is common (the wildtype) but only weakly selected against when *CD* is common, causing the fitness surface to fall faster near *cd* than near *CD* when the toxin load is stronger.

In a spatial context (explored below), we expect genetic constructs with higher thresholds (larger *s*_*p*_, smaller *s*_*t*_) to be less likely to expand into neighbouring populations, because invasion will not occur unless a sufficient number of transgenic haplotypes have been introduced. However, shifting the unstable equilibrium too far to the transgenic haplotype makes it challenging to establish the construct in the first place and makes reversion to the wildtype state more likely due to drift and incoming wildtype migrants from neighbouring populations. Using different toxin-antidotes and/or payloads can, in principle, allow these benefits and risks to be adjusted.

### 2.3 Invasion analysis

We next perform an invasion analysis to determine the force of drive *δ*_*c*_ needed for the transgenic alleles *C* and *D* to spread. By force of drive, we refer to the level of drive currently experienced by the population, which is the drive strength *δ* times the frequency of individuals bearing the driving allele *B* among heterozygotes at the cargo loci (*Cc* or, equivalently, *Dd*). We first consider whether the fitness-valley construct could spread if *δ*_*c*_ were constant at its current level. Assuming that the daisy quorum drive is introduced altogether (as *ABCD/ABCD* homozygotes or *ABCD/abcd* heterozygotes), the first heterozygotes that appear at loci **C** and **D** initially bear the driver allele *B*, so *δ*_*c*_ = *δ*. The genetic association between loci **B** and **C** or **D** decreases over time due to recombination, however, weakening the drive force experienced by the fitness-valley construct over time, as discussed in S1 Appendix, section S1.4.

For a fixed drive force *δ*_*c*_, the invasion fitness of the payload cargo (loci **C** and **D**) equals the larger of the two eigenvalues in a stability analysis, λ_*L*_:

$$\begin{array}{c} \lambda_{L}=\text{max}\left[\left(1+\delta_{c}\right)\left(1-s_{p}\right)\left(1-s_{t}\right),\left(1+\delta_{c}^{2}-r{\left(1-\delta_{c}\right)}^{2}\right)\left(1-s_{p}\right)\right]. \end{array}$$ (4)

The first of the eigenvalues describes the rate of spread of either allele *C* or *D* on its own. The second describes the spread of the combined *CD* haplotype, which is slowed by recombination.

**Fig 3:**
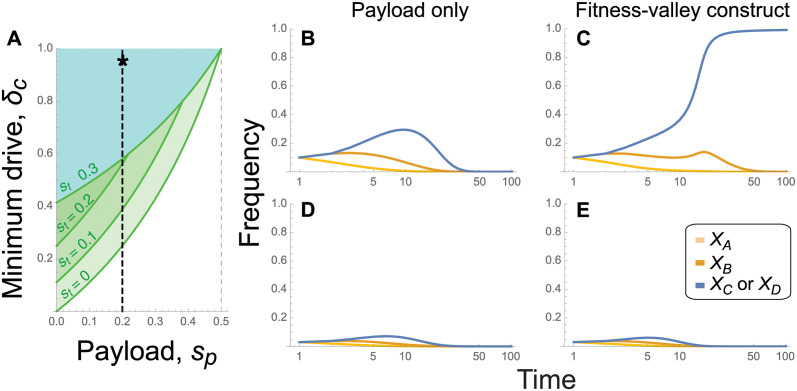
Necessary conditions for spread of a cargo and example dynamics. Panel **(A)** gives the minimum amount of drive, $\delta_{c}^{*}$, required for spread of the cargo when rare and introduced with the driver allele according to Eq (5), illustrated for different toxin loads (*s*_*t*_ by color) and payloads (*s*_*p*_ on the horizontal axis). Remaining panels show the temporal dynamics for a daisy quorum chain pushing a payload of *s*_*p*_ = 0.2 (dashed line in panel **(A)**). The payload is carried by a single-locus cargo allele *C* without toxicity (*s*_*t*_ = 0) in panels **(B),(D)** or by a fitness-valley construct with alleles *C* and *D* carrying toxins and their antidotes (*s*_*t*_ = 0.9) in panels **(C),(E)**. The panels are initialized with **(B)** gametes *ABCd* at frequency *f*_0_ = 0.1, **(C)** gametes *ABCD* at frequency *f*_0_ = 0.1, **(D)** gametes *ABCd* at frequency *f*_0_ = 0.03, **(E)** gametes *ABCD* at frequency *f*_0_ = 0.03. Other parameters are: *δ* = 0.95, *s*_*d*_ = 0.1, and *R* = *r* = 0.5.

Focusing on unlinked loci ($r=\frac{1}{2}$), a necessary condition for the cargo to spread when rare (λ_*L*_ > 1) is that drive force be stronger than the critical value, $\delta_{c}^{*}$:

$$\begin{array}{c} \delta_{c}>\delta_{c}^{*}=\text{min}[\frac{1}{\left(1-s_{p}\right)\left(1-s_{t}\right)},\sqrt{\frac{2}{1-s_{p}}}]-1 \end{array}$$ (5)

(Fig 3A). With a low toxin load (*s*_*t*_), the first criterion is easier to satisfy, and we expect the alleles to spread individually (green regions in Fig 3A). With a high toxin load, however, only the combined haplotype *CD* will spread initially (blue region in Fig 3A), as long as drive is strong enough to counter the haplotype being broken down by recombination.

Dominant alleles carrying a very strong payload that reduces fitness by more than half, *s*_*p*_ > 1/2, can never invade with a dominant payload (Fig 3A), as they would require a stronger drive force than is possible (*δ*_*c*_ > 1, Eq 5). When the payloads at loci **C** and **D** have multiplicative or recessive effects, however, a construct bearing an even higher payload than *s*_*p*_ = 1/2 can spread initially, but the construct does not reach fixation because individuals such as *Cd/cD* express a lower payload while also avoiding the toxin load and so have higher fitness than *CD/CD* homozygotes (see S1 Appendix, sections S1.5 and S1.6).

**Fig 4:**
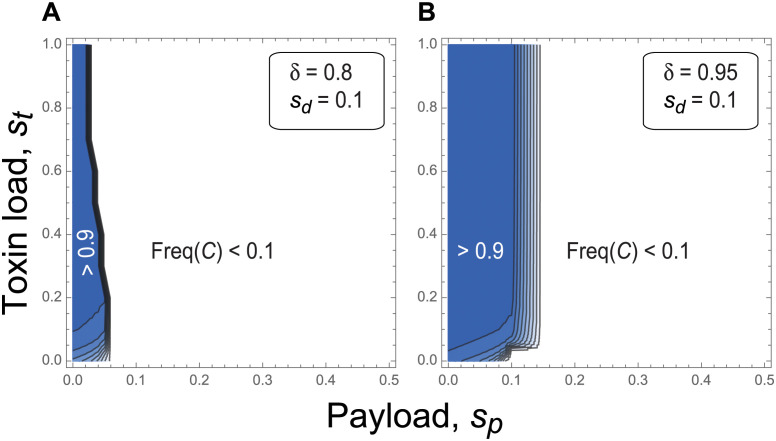
Parameters allowing fitness-valley crossing in the four-locus model of daisy quorum drive. The frequency of the fitness-valley construct *C* (or equivalently *D*) at generation *t* = 100 is illustrated across all values of the toxin load and payload where initial spread is possible. Results at *t* = 500 are similar. Panel **(A)**: Weaker drive of *δ* = 0.8. Panel **(B)**: Stronger drive of *δ* = 0.95. Other parameters are: *f*_0_ = 0.05, *s*_*d*_ = 0.1, and *R* = *r* = 0.5.

Importantly, the above equations treated the drive force as fixed. As time passes, recombination breaks the initial disequilibrium between the driving allele *B* and the driven alleles (*C* and *D*) and eventually selection eliminates the daisy chain allele *B* because of the drive load. Whether the initial push from the daisy chain is sufficient to fix the fitness-valley construct is evaluated numerically by iterating the full four-locus recursion equations (Figs 3 and 4).

First consider Fig 3B and 3D, which illustrate a daisy chain driving a constant “cargo” load, for comparison, without a toxin-antidote (no fitness valley). This cargo is modeled as a third locus **C’** that only contains a payload (no toxin load), as is often considered with daisy-chain drives. In all cases, drive is initially strong enough to satisfy Eq 5 (star in Fig 3A), and the cargo rises in frequency (blue curves). The first daisy chain allele *A* is not driven and declines over time (light orange). The second daisy chain allele *B* initially rises due to drive by *A* but then declines following the loss of *A*. Once drive is exhausted, a constant cargo is counterselected because of the payload (blue curve in Fig 3B and 3D). While a longer daisy chain could be used to drive the cargo to such a high frequency that it would be expected to fix in any finite population, such constructs would be prone to loss after drive has exhausted once wildtype alleles, which lack the payload, are reintroduced by migration or mutation.

Next, consider daisy quorum drive, where the daisy chain drives a fitness-valley construct (Fig 3C and 3E). Alleles *C* and *D* rise in frequency and fix as long as the initial release frequency, *f*_0_, is high enough that drive can push the construct past the fitness valley before exhausting (compare blue curve in Fig 3C with *f*_0_ = 0.1 to that in Fig 3E with *f*_0_ = 0.03). Longer daisy-chains can drive the cargo to fixation starting from even lower initial frequencies (e.g., *f*_0_ = 0.02 is sufficient with a daisy chain of three loci, see *Mathematica* file on Zenodo doi:10.5281/zenodo.10904198). Thus, by coupling the fitness-valley construct to a transient daisy-chain drive, alleles *C* and *D* can establish at a far lower initial release frequency than predicted for the fitness-valley construct on its own (introducing only *CD*), which requires a starting frequency of over 0.36 for the parameters considered in Fig 3 (see *Mathematica* file on Zenodo (doi:10.5281/zenodo.10904198).

Fig 4 explores the parameter combinations that allow successful establishment of the fitness-valley construct. The full four-locus recursion equations are iterated for 100 generations, at which point the frequency of allele *C* (or equivalently *D*) is calculated and shown. Interestingly, establishment of the fitness-valley construct does not depend much on the toxin load (y axis), because drive is strong enough to spread the *CD* construct past the fitness valley created by the toxin. By contrast, establishment depends critically on the payload (x axis). Higher payloads reduce the speed at which the construct spreads when first introduced (Eq 4) and increase the position of the fitness valley (separatrix) experienced once drive is exhausted (S2 Fig). Consequently, stronger drive or longer drive chains are needed to push more costly payloads to high frequency (e.g., compare Fig panel 4B to 4A).

### 2.4 Spatial spread of daisy quorum drive in a discrete environment

We now consider the drive system in a spatial context, first with discrete spatial patches and then in continuous space, asking when spatial confinement is possible with daisy quorum drive. To begin, we investigate a linear stepping-stone model of *M* = 101 interconnected populations with migration rate *m* between adjacent patches. Initially, we consider populations of large and constant size. Migration is followed by dynamics within each patch, as described in section 2.2. The frequency of gametes of type *i* after migration for a non-boundary population *j* is given by: $p_{i}^{j^{\prime }}=\left(1-m\right)p_{i}^{j}+\frac{1}{2}mp_{i}^{j-1}+\frac{1}{2}mp_{i}^{j+1}$. The boundary populations only give and receive migrants from the interior: $p_{i}^{0^{\prime }}=\left(1-\frac{1}{2}m\right)p_{i}^{0}+\frac{1}{2}m\;p_{i}^{1}$ and $p_{i}^{M^{\prime }}=\left(1-\frac{1}{2}m\right)p_{i}^{M}+\frac{1}{2}m\;p_{i}^{M-1}$. Using the full four-locus recursions, we explore the spread of daisy quorum drive through migration among patches.

**Fig 5:**
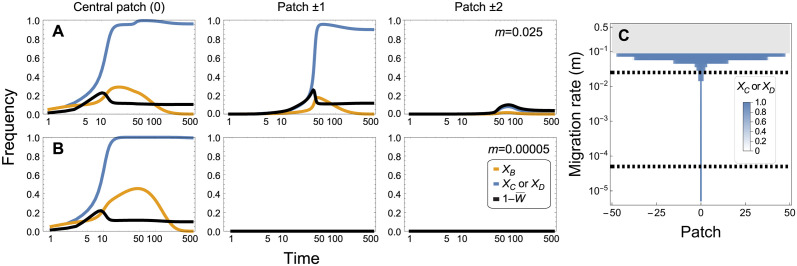
Dynamics of spread of a payload for different migration rates. The dynamics in the central patch and the two adjacent patches (panels from left to right) are shown following the introduction of chromosomes bearing *ABCD* alleles at time 0 into the central patch at an initial frequency of *f*_0_ = 0.05. Broader expansion to neighbouring populations occurs with the higher migration rate in panel **(A)** (*m* = 0.025) than with the lower migration in panel **(B)** (*m* = 0.00005). As predicted, the drive eventually disappears from the system due to the drive load *s*_*d*_ = 0.02 (orange curves). Panel **(C)** shows the frequency of the payload alleles, *X*_*C*_ or *X*_*D*_, at 1000 generations (blue shading) across all patches (x axis) for a variety of migration rates (y axis). In the gray area of panel **(C)**, migration into the central patch is high enough to swamp the engineered constructs, causing no spread to occur anywhere (dashed lines represent the migration rates used in panels **(A)**,**(B)**). Other parameters are: *δ* = 0.9, *s*_*t*_ = 0.9, *s*_*p*_ = 0.1, and *R* = *r* = 0.5.

The migration rate *m* is a crucial parameter determining the expansion of the fitness-valley construct across interconnected populations (Fig 5). For moderate to low migration rates (*m* < 0.02), the construct spreads to high frequency locally but remains spatially restricted, as intended. By contrast, if migration is so high that the population is nearly panmictic (*m* > 0.1 in this one-dimensional stepping stone model), the construct fails to spread due to gene swamping, causing the frequency at the point of introduction to fall below the threshold needed for invasion (grey area in Fig 5C). Intermediate migration rates pose the greatest risk of spread (*m* roughly between 0.02 and 0.1), because migration is not too high to swamp the construct but high enough to lead to broad expansion of the construct over space (blue horizontal stripe in Fig 5C). We expect that the exact boundaries between these regions depend on the spatial structure under consideration (e.g., gene swamping leading to loss of the construct may occur more often in a fully interconnected island model, for a given total immigration rate per patch, because the stepping-stone model considered here allows the construct to accumulate in adjacent patches, which reduces swamping).

These results are consistent with the simulations of a two-locus fitness-valley construct without drive by Dhole et al. [8]. Although their fitness regime differed (see S1 Appendix, section 1.8, and S6 Table), these authors also found that spread to adjacent populations occurred more often when migration rates were intermediate (their Fig 5b2). The main difference is that, as expected, a lower introduction frequency is needed with daisy quorum drive (here) than in their simulations of a fitness-valley construct without drive. Dhole et al. also explored one-locus fitness-valley (underdominant) constructs [8], which were less prone to spread to adjacent populations but required even higher initial frequencies (high threshold, lower risk of expansion), as well as a daisy chain on its own, which exhibited the opposite characteristics (low threshold, higher risk of expansion; see their Fig 5).

**Fig 6:**
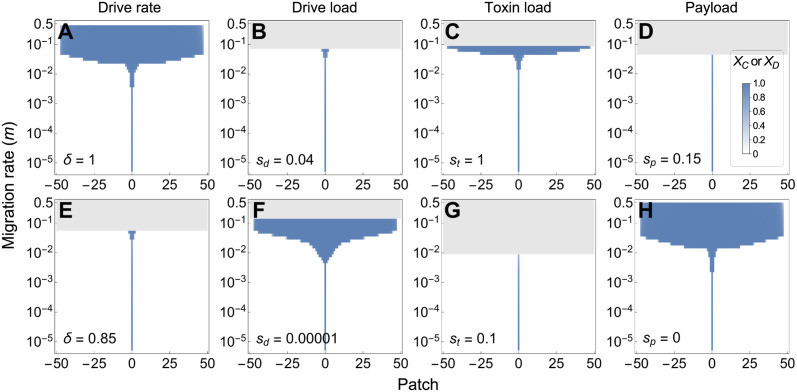
Illustration of the effect of changing a single parameter on the results shown in Fig 5C. The frequency of the payload is shown in blue after 1000 generations when *ABCD* gametes are introduced into the center patch (patch 0) at frequency *f*_0_ = 0.05. Patches are linearly connected and exchange migrants every generation at a rate *m*. Panels increase (top) or decrease (bottom) a single parameter: **(A)**,**(E)** drive rate *δ*, **(B)**,**(F)** drive load *s*_*d*_, **(C)**,**(G)** toxin load *s*_*t*_, and **(D)**,**(H)** the payload *s*_*p*_. Unless specified, parameters are: *δ* = 0.9, *s*_*d*_ = 0.02, *s*_*t*_ = 0.9, *s*_*p*_ = 0.1, and *R* = *r* = 0.5.

Given a particular migration rate *m*, the power of the daisy chain combined with the location of the separatrix determine the extent of expansion into neighbouring populations. Increasing the drive rate *δ* (Fig 6A versus Fig 6E) or lowering the drive load *s*_*d*_ (Fig 6F versus Fig 6B) results in a stronger daisy chain that is more likely to spread the fitness-valley construct to more populations, as would lengthening the daisy chain *n* (S1 Fig). By contrast, raising the payload *s*_*p*_ reduces the risk of spread (Fig 6D versus Fig 6H). Interestingly, decreasing the toxin load *s*_*t*_ reduces the risk of spread of the payload to neighbouring populations, even under high migration rates, despite making the fitness valley shallower (Fig 6C versus Fig 6G). This is consistent with our analysis of the two-locus fitness-valley construct on its own (section 2.2), where we noted that the lower the toxin load, the closer the fitness valley (separatrix) is to fixation of the construct *CD* and the higher the introduction frequency must be to cross the fitness valley (S2 Fig). Thus, somewhat counterintuitively, a lower toxin load provides a higher degree of safety in isolating the fitness-valley construct from neighbouring patches once the daisy-chain drive has exhausted itself (this assumes that each toxin is neutralized by a single antidote allele, as in Table 1).

Finally, we considered populations of finite size, where the daisy quorum drive could affect the number of surviving individuals. Stochastic simulations confirm that the above results are robust to finite population sizes in cases where the population persists (S7 Fig), corresponding to population modification. In cases where the payload drives the local population extinct, corresponding to a case of complete population suppression, recolonization by wildtype individuals eventually occurs from neighbouring populations (S8 Fig). Daisy quorum drive, like other drive systems, is thus only transiently effective in the face of gene flow when the goal is complete population suppression in a local area.

### 2.5 Spatial spread of daisy quorum drive in a continuous environment

We now ask whether the construct can be confined even if space were continuous and under what conditions. We continue to use the dynamics described in section 2.2 for the change per unit time for each gamete frequency prior to migration, coupling these dynamics with movement across space, considering small intervals of time and space to approximate continuous dynamics (see S3 Appendix for details). When considering a homogeneous environment, population densities and diffusion rates are held constant over time and space (with diffusion rate D=0.2 per time unit and spatial unit).

**Fig 7:**
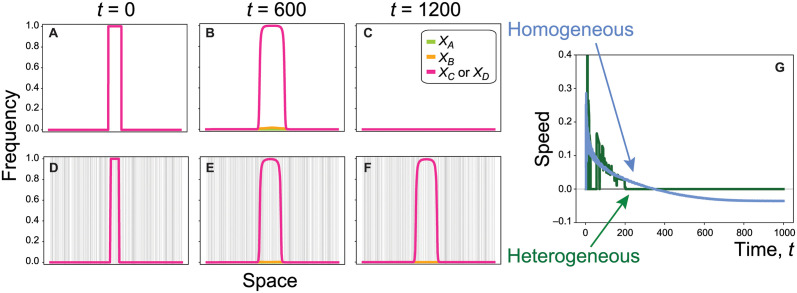
Comparing the spread of introduced genotypes across homogeneous or heterogeneous landscapes. The first three panels in each row represent allele frequencies in one-dimensional space at times *t* = 0, 600, 1200. The rows illustrate simulations with homogeneous distances between adjacent sites (top row) or heterogeneous distances (bottom row, with step sizes determined by randomly choosing 400 boundaries across the spatial domain). The last panel describes the speed of the cargo wave measuring the frequency of either *C* or *D* (i.e., the speed of the pink curve in previous panels, in spatial units per unit time), either in a homogeneous (blue) or heterogeneous (green) environment. (In the heterogeneous case, we measure the speed of the left side of the wave.) The payload is *s*_*p*_ = 0.35. This payload value was chosen so that the wave collapses in a homogeneous environment (with a negative asymptotic speed; top row) but can first expand due to drive and then be stably maintained with spatial heterogeneity (bottom row). Other parameters: *δ* = 0.9 (drive rate), *s*_*d*_ = 0.02 (drive load), *s*_*t*_ = 0.9 (toxin load), *R* = *r* = 0.5 (recombination rate), D=0.2 (diffusion rate), and *L* = 200 (length of the spatial domain).

In a continuous and homogeneous environment, a daisy quorum drive system does not reach a stable and spatially-restricted equilibrium. For example, the top row of Fig 7 illustrates the spread from a central introduction zone across a homogenous one-dimensional spatial region, using a reaction-diffusion model. The construct spreads initially due to the daisy chain drive (Fig 7A and 7B), but then contracts (Fig 7C) with the wavefront slowing in speed and eventually reversing (blue curve, Fig 7G).

Thus, when space is homogeneous, either the construct is driven outward in an expanding wave or the wave collapses in on itself. In either case, the wave speed approaches a non-zero constant over time. In S11 Fig, we explore the asymptotic wave speed across a range of possible spatial step sizes, from a nearly uniform distribution of individuals over space (“continuous”) to an increasingly clumped distribution (“discrete”) by increasing the size and distance between patches in a stepping stone model. Only in sufficiently patchy environments is the fitness-valley construct expected to remain in place, as found by Barton with one-locus underdominance [44].

Importantly, in the continuous space model, the asymptotic wave speed is the same whether or not the driver alleles *A* and *B* are initially present because drive is eventually exhausted. Despite the difference in asymptotic wave speed between discrete and continuous space models, spatial spread of the fitness-valley construct is more extensive in either case if the toxin load is more severe (higher *s*_*t*_) or the payload milder (lower *s*_*p*_), either of which lower the threshold for spread of the construct (S2 Fig).

To avoid spread of the transgenic constructs across environments that are spatially continuous, we recommend that the toxin load be light enough and the payload heavy enough to ensure that the fitness-valley construct is not expected to spread unless driven (i.e., it has a negative asymptotic wave speed, S11 Fig). As discussed in S1 Appendix, section 1.7, fitness valleys created by haploinsufficient gene swapping have high thresholds (above 50%) as long as alleles *C* and *D* carry a payload, which we expect also prevents their spatial expansion.

While constructs with negative wave speeds would eventually be expected to disappear in a continuous environment where density and dispersal rates are homogeneous over space, we find that such constructs can be stably maintained if there is spatial heterogeneity. The bottom row in Fig 7 illustrates such an example, where grey vertical bars represent inhospitable regions (patch edges). The construct initially spreads due to drive but eventually is stabilized in space. Regions that support fewer individuals or that are hard to traverse effectively act as barriers, stabilizing the daisy quorum drive system in space. While some wild-type individuals can enter a region where the construct has stabilised, they are never numerous enough to trigger an invasion. Similar behaviour has been observed in the one-locus underdominant system studied by Barton [44].

In summary, to avoid indefinite spread within populations that are continuously distributed over space, fitness-valley constructs could be designed with negative asymptotic wave speeds (S11 Fig). Daisy quorum drive would then transiently spread the fitness-valley construct until the drive alleles disappear. The benefits of the payload carried by the fitness-valley construct are then only expected in a limited spatial region, which shrinks over time unless stabilized at locations where movement is impeded.

### 2.6 Comparison to other spatially restricted drive constructs

Several other transgenic designs have been proposed that combine various features of drive and fitness costs to restrict spatial expansion. Here we briefly summarize four of these designs and compare them to daisy quorum drive. Before doing so, we briefly discuss differences between the daisy quorum drive considered here and the design proposed by [7].

While we were developing the design described in this paper, daisy quorum drive was proposed by [7] using different components. Rather than generating a fitness valley using a toxin-antidote system (Table 1), they proposed swapping two haploinsufficient genes. Using the terminology of the current paper, individuals without a total of two copies of either allele *c* or *D* (which carries out the function of locus **C**) or without two copies of *d* or *C* (which carries out the function of locus **D**) would suffer the cost of haploinsufficiency at these genes. The resulting fitnesses are summarized in S5 Table, including a payload for alleles *C* and *D*. In the design of [7], the gRNAs are carried by the driver loci, but CRISPR is carried by the fitness-valley genes **C** and **D**, so that the CRISPR gene would remain even after drive is exhausted, which is avoided here. Otherwise, similar results are observed when the fitness valley is created by two-locus haploinsufficient gene swapping or the toxin-antidote system (see *Mathematica* file on Zenodo doi:10.5281/zenodo.10904198). The most important difference is that the threshold required for spread of the haploinsufficient alleles *C* and *D* is either at (if *s*_*p*_ = 0) or above (if *s*_*p*_ > 0) 50%. Thus, creating fitness valleys via haploinsufficient gene swapping has the benefit of a high threshold. This higher threshold requires stronger drive to push the *CD* construct across the fitness valley and to high frequency (compare S6 Fig to Fig 4) and is expected to reduce rates of expansion over space.

Split drive killer-rescue systems (SDKR) add homing split drive [48] to a killer-rescue system, which also creates a fitness-valley [37]. Here the driver allele (*B*) carries Cas9 and rescues individuals from the toxic “killer” effects carried by the driven allele *A*. In turn, allele *A* carries the gRNA, which cleaves the wild-type allele at locus **A** (splitting refers to the genetic separation of Cas9 from the gRNAs). Gene drive favours the rise in frequency of allele *A*, which in turn selects for allele *B* to rescue the killer effects of *A*. Because each allele carries a cost on its own, but the killer phenotype is rescued in *AB* individuals, SDKR generates a fitness valley, requiring a higher introduction frequency to spread and limiting expansion into neighboring populations (explored using a two-deme model by Edgington et al. [48]). Furthermore, SDKR was shown to be relatively robust to fitness costs of the transgene alleles and homing rates. Because drive persists in the SDKR system, however, CRISPR-Cas9 elements are expected to remain in the population for prolonged periods of time, and SDKR remains susceptible to the spread of alleles resistant to Cas9 generated by NHEJ and other mechanisms, limiting its persistence. Compared to daisy quorum drive, SDKR also requires a high introduction frequency.

HD-ClvR systems are composed of homing (H), daisyfield (D), and cleave-and-rescue (ClvR) gene drives [49]. The daisyfield distributes gRNA throughout the genome, at separate loci from the ClvR construct carrying Cas9; thus, mating with wildtype individuals dilutes the number of gRNA carried by the same individuals as Cas9, limiting the time period over which drive acts. The gRNA guides cleavage at both the ClvR construct (allowing homing) and another haploinsufficient gene; ClvR itself carries a functioning allele of that haploinsufficient gene, so only cells carrying the ClvR construct survive following cleavage. This system is less sensitive to NHEJ and resistance evolution, because resistant alleles at the ClvR site will not compensate for cleavage of the haploinsufficient target and resistance alleles created by NHEJ are unlikely to function at the haploinsufficient gene (as recently shown in a split drive system in *Drosophila*, [50]). Because the dilution effect of the daisyfield and fitness costs lead to the loss of the constructs, additional individuals carrying the gRNA elements need to be introduced to sustain the constructs when and where intended [49]. Like a daisy quorum system, initial release frequencies can be small (due to drive), but HD-ClvR requires repeated release to maintain the population suppression or modification, and the CRISPR-Cas9 elements remain in the population.

Another approach to restricting gene drives in space involves tethered homing gene drive, where a fitness-valley construct is first introduced that limits (“tethers”) where drive can occur. One design combines a two-locus toxin-antidote system with a homing split drive [51]. The two toxin-antidote loci **A** and **B** induce a fitness valley and carry Cas9, but the gRNA and its target are at a separate homing locus **C**, which carries the payload. Releasing the toxin-antidote system at high enough frequency in a local region then enables drive to high frequency of allele *C*. Tethered homing constructs are less able to spread across space because of the fitness-valley and payload, causing each construct to be selected against when rare. Nevertheless, spatial expansion can occur for the two-locus toxin-antidote system, as we have shown here. Compared to daisy quorum drive, tethered homing gene drive requires high initial release frequencies (to get *A* and *B*, which are not driven, to spread across their fitness valley). It also retains the CRISPR-Cas9 homing system indefinitely.

Finally, a very different approach to spatial confinement has been proposed that takes advantage of genetic differences among populations. For example, [52] considered a double drive design causing sex-ratio distortion, where a CRISPR-Cas9 element is introduced onto the Y chromosome targeting and editing an X-linked gene into a form that is dominant lethal or infertile when inherited by daughters. A second X-shredder construct is introduced on an autosome to destroy X-bearing sperm, creating segregation distortion that favours the spread of the Y construct. To confine the two constructs in space, the authors propose taking advantage of genetic differences at the autosomal shredder or its X-linked gene target, so that only local populations are susceptible. Again, like daisy quorum drive, this design allows constructs to be introduced only once in small numbers. By contrast, CRISPR-Cas9 is retained in the population, with greater potential for the evolution of resistance, as well as the evolution of recognition to the genetic variants present at other locations, allowing expansion outside of the intended region (see discussion of various design considerations in [52]).

## 3 Discussion

While gene drive has the potential to ameliorate many challenges facing humanity [1], mechanisms based on CRISPR-Cas9 homing drives face two major hurdles: concerns about spread to neighbouring populations by migration (or to closely related species by hybridization) and the rapid evolution of target sites that resist cutting by Cas9. We thus explored a gene-drive design system that transitions over time from homing-based gene drive, which has a low threshold frequency for spread and is especially susceptible to spread beyond the target area, to a fitness-valley construct, with a high threshold frequency that is more readily contained. This design, called “daisy quorum drive” by Min et al. [7], couples a daisy-chain technique proposed by Noble et al. [4], which limits the temporal extent of gene drive, and a two-locus fitness-valley construct, which helps to restrict the construct from expanding across space because it is disfavoured when rare in the absence of drive [8].

Here, we have developed and analysed the first model of the full daisy quorum drive system. We find that this design can lead to the local spread and maintenance of a payload that alters a target population in discrete environments, while limiting further expansion because the driver alleles are eliminated over time. Importantly, daisy quorum drive provides a method by which populations can be modified without requiring a high introduction frequency, which is logistically or economically unfeasible for many species (including non-model species that are challenging to breed in captivity and threatened species from which large samples cannot be taken to establish breeding populations). Furthermore, because drive is transient, all CRISPR-Cas9 elements are removed by selection from the population, so that subsequent spread to other populations does require a high frequency, reducing risk. The fitness-valley construct remains spatially restricted as long as it is built with a light enough toxin load and a heavy enough payload to avoid spread through more homogeneous environments (Figs 5, 6 and 7). In agreement with others [9, 44, 45], we find that spatial containment occurs readily when populations are distributed patchily over space but is more challenging when populations are continuously and homogeneously distributed over space. Designing constructs that contract over time in spatially homogeneous environments (low *s*_*t*_ and high *s*_*p*_) thus provides a margin of safety with respect to containment. As observed in Fig 7D–7F, such constructs could provide benefits for a prolonged period even in such homogenous environments, whenever the collapsing wave becomes caught in regions of low density or dispersal.

Previous models of homing-based gene drives have raised substantial concerns about the rise of resistance (e.g., alleles no longer recognized by the gRNA following NHEJ or mutation), which prevents the drive from reaching fixation in the target population and can lead to the loss or partial loss of the engineered constructs over time [19]. Here we have not taken resistance alleles into account because the daisy-chain drive system, as used in daisy quorum drive, stops driving and declines in frequency soon after its introduction, before resistance is likely to rise substantially in species with efficient homology-based repair [4]. This attribute differs from the daisy-chain drive system on its own, where the drive construct has to be repeatedly introduced to maintain the cargo over time [4]. Longer daisy chains can also be used to extend the time before loss, even when error-prone repair generates resistant alleles [4]. Daisy chains where the gRNA for each locus is contained in the construct for that locus itself reduce the rate of appearance of resistant alleles by preventing cutting when the construct is absent (an “indirect daisy-chain” design) [34]; this design could be extended into the fitness-valley construct as well, but then gRNA elements would persist. We caution, however, that the two-locus fitness-valley construct is susceptible to mutations that decrease or compensate for the payload, which would affect the long-term persistence and spread of the fitness-valley construct. Mutations that reduce the toxin load are expected to have less effect because the toxin load is transient and disappears if the fitness-valley construct *CD* fixes, as expected if the payload is dominant. Unlike previous scenarios considered [8, 9], there is a benefit to having the transgenic alleles *C* and *D* carry a dominant payload *s*_*p*_ (as in Table 1), because the construct is then expected to fix and resist the spread of mutations that reverse the payload in only one allele, which is neutral while the payload remains at the other alleles. Despite these advantages of a dominant payload, recessive and multiplicative payloads are also feasible (sections S1.5 and S1.6), although such constructs might be less long-acting because of the selective benefits of mutations that inactivate the toxin.

Future modelling work would benefit from explicitly incorporating resistance mutations at each of the loci. It would also be valuable to explore further the impact of daisy quorum drive on population size, especially if population suppression is the goal (most of our results assumed a constant population size, except for the discrete patch simulations of S7 and S8 Figs. Demographic effects can have major consequences for the conclusions of gene drive models [8, 53–55]. To accurately predict real-world dynamics before any field trial, it will be important to accurately model density-dependence in the species of interest and how growth rates, competitive interactions, and population density are affected by the fitness costs of the various components of the daisy quorum drive. All else being equal, we expect that localized declines in population density caused by daisy quorum drive would inhibit expansion over space. This is consistent with our discrete-patch stochastic simulations, where we observed that the spread of a daisy quorum drive was more limited in space and time when it strongly suppressed local population sizes (compare S7 to S8 Figs).

While gene drives have the potential to solve many ecological challenges, not all consequences of triggering a gene drive can be foreseen. Models like the ones presented here are an important first step, but they only serve as a rough guide. Lab-based research is needed to determine the feasibility of developing daisy quorum drive with the necessary features, combining daisy gene drive with a fitness-valley construct. Controlled experiments are needed to determine the probability that mutations reverse the payload (eliminating the intended benefits of the release program) and the risk of unintended side effects, such as off-target cleavage, off-target drive, or biological interactions with the toxin-antidote system. Models tailored to the genetic and ecological details of a particular system are also needed, incorporating experimentally measured parameters and natural history details. Simultaneously, fulsome and community-guided evaluations of potential harms and assessments of risk [46] are essential to avoid negative consequences and obtain informed consent before release of gene drives in the wild [1].

In summary, we explored the potential for daisy quorum drive to combine two desirable features: a daisy chain involving homing-based gene drive that allows rapid spread from a low introduction frequency but then disappears, and a fitness-valley construct that is less prone to expand across space. We find that combining these features provides a potentially valuable option for population modification, requiring a low initial release size, allowing rapid initial spread, persisting locally, but having little to no long-term movement over space once the daisy chain becomes exhausted. Furthermore, all CRISPR-Cas9 elements are counterselected and expected to disappear once drive is exhausted.

## Supporting information

S1 Appendix Daisy quorum drive in a single population. (PDF)

S2 Appendix Individual-based simulations with discrete patches. (PDF)

S3 Appendix Numerical simulations in continuous space. (PDF)

S1 Fig Maximum frequency of the last element in an *n* length daisy-chain construct in a single population. A daisy chain of size *n* is designed by repeating locus **A** in Fig 2 for **A_1_**,…, **A_*n*−1_**, each carrying a gRNA targeting the next locus in the chain. All elements of the daisy-chain carry a drive load *s*_*d*_ = 0.05. The frequency of the *n*^th^ transgenic allele is denoted by *X*_*n*_. Other parameters are *δ* = 1, *R* = 0.5.
(EPS)

S2 FigTernary plot illustrating the separatrix (the boundary separating basins of attraction to different fixed points) for the two-locus fitness-valley construct.The construct creates a toxin load of *s*_*t*_ = 0.1 in panel **(A)** or *s*_*t*_ = 1.0 in panel **(B)** and a payload of *s*_*p*_ = 0.0, 0.2 and 0.8 (green curves). Locus **C** and **D** recombine freely ($r=\frac{1}{2}$). The dashed curves are example dynamics for *s*_*p*_ = 0.2.(EPS)

S3 Fig The dynamics of the full four-locus daisy quorum drive. The toxin load is low in panels **(A)**,**(B)** (*s*_*t*_ = 0.1) and high in panels **(C)**,**(D)** (*s*_*t*_ = 0.9). The frequency of the toxic allele *C* (equivalently *D*) is shown in blue, with *ABCD* introduced at frequency *f*_0_ = 0.015 (panels **(A)**,**(C)**) or *f*_0_ = 0.02 (panels **(B)**,**(D)**). The toxin-antidote alleles *C* and *D* together rise to fixation (crossing the fitness valley) only when started at a high enough initial frequency to suppress each other’s toxicity (panels **(B)**,**(D)**). The frequency of the drive phenotype (including both *BB* and *Bb* genotypes) is shown among *Cc* heterozygotes (equivalently *Dd* heterozygotes; solid orange) and in the full population (dashed orange). The green curve shows the position of the unstable equilibrium of the fitness-valley construct, given the current frequency of the drive phenotype in *Cc* (or in *Dd*) heterozygotes, with the star indicating the position once drive has disappeared. Parameters: *δ* = 0.9, *R* = *r* = 0.5, *s*_*d*_ = 0.02, *s*_*p*_ = 0.1.
(EPS)

S4 FigEquilibria with multiplicative expression of the payload (S3 Table).The coloured curves indicate the internal unstable (green) and stable (blue) equilibria for different payload values *s*_*p*_, either with a low toxin load (*s*_*t*_ = 0.1, top curves) or a high toxin load (*s*_*t*_ = 1, bottom curves). For a given payload, *s*_*p*_, starting to the right of the green point will lead the system to approach the blue point with the same color saturation (*s*_*p*_ value). Recombination rate *r* is $\frac{1}{2}$, and the dashed curve indicates when there is no linkage disequilibrium between the two loci. If the payload is too strong relative to the toxin load, the internal equilibria become complex, as denoted by the * (for *s*_*p*_ ≥ 0.11 with *s*_*t*_ = 0.1 and for *s*_*p*_ ≥ 0.73 with *s*_*t*_ = 1), at which point only the wildtype equilibrium is stable, and the fitness-valley construct is lost. Recombination rate *r* is $\frac{1}{2}$, and the dashed curve indicates when there is no linkage disequilibrium between the two loci.(EPS)

S5 FigEquilibria with recessive expression of the payload (S4 Table).The coloured curves indicate the internal unstable (green) and stable (blue) equilibria for different payload values *s*_*p*_, either with a low toxin load (*s*_*t*_ = 0.1, top curves) or a high toxin load (*s*_*t*_ = 1, bottom curves). For a given payload, *s*_*p*_, starting to the right of the green point will lead the system to approach the blue point with the same color saturation (*s*_*p*_ value). If the payload is too strong relative to the toxin load, the internal equilibria become complex, as denoted by the * (for *s*_*p*_ ≥ 0.098 with *s*_*t*_ = 0.1 and for *s*_*p*_ ≥ 0.71 with *s*_*t*_ = 1), at which point only the wildtype equilibrium is stable, and the fitness-valley construct is lost. Recombination rate *r* is $\frac{1}{2}$, and the dashed curve indicates when there is no linkage disequilibrium between the two loci.(EPS)

S6 Fig Parameters allowing fitness-valley crossing in the four-locus model of daisy quorum drive when *C* and *D* carry swapped versions of two haploinsufficient genes [7]. The frequency of the fitness-valley construct *C* (or equivalently *D*) at generation *t* = 100 is illustrated across all values of the toxin load and payload where initial spread is possible. Only in the darker regions (constructs above 50%) does the construct remain at *t* = 500. Panel **(A)**: weaker drive with *δ* = 0.8; panel **(B)**: stronger drive with *δ* = 0.95. Other parameters are: *f*_0_ = 0.05, *s*_*d*_ = 0.1, and *R* = *r* = 0.5.
(EPS)

S7 Fig Population modification with daisy quorum drive in finite populations. Black dashed curve is the total population size at time *t*, blue solid curve represents the number of individuals carrying the cargo allele *C* (equivalently *D*), and orange solid curve represents the number of individuals carrying the driver allele *B*. In this case, fertility is set to *F* = 1.2 and the payload to *s*_*p*_ = 0.1, so that subpopulations can persist even if the fitness-valley construct fixes. Each patch has a carrying capacity of *K* = 10000. Rows have a migration probability between adjacent patches of **(A)** *m* = 0, **(B)** *m* = 0.05, **(C)** *m* = 0.1 from top to bottom. Drive is released only in the central patch at frequency *f*_0_ = 0.05 (left panels). The solid curves represent the median of 50 replicates, and the shaded regions the first and third quartiles.
(EPS)

S8 Fig Population suppression with daisy quorum drive in finite populations. Identical to S7 Fig but with fertility of *F* = 1.05, which is too low for a suppopulation to replace itself once the fitness-valley construct with a payload of *s*_*p*_ = 0.1 has fixed.
(EPS)

S9 FigSpread of introduced genotypes across one-dimensional continuous space that is homogeneous.The first three panels in each row represent allele frequencies in space at times **(A,E)** *t* = 0, **(B,F)** *t* = 300, and **(C,G)** *t* = 600. The right-most panels **(D,H)** show the speed of the *X*_*C*_ wave (or equivalently *X*_*D*_ wave) as a function of time. The rows illustrate simulations with a low payload (top row, *s*_*p*_ = 0.1) or a high payload (bottom row, *s*_*p*_ = 0.5). The simulations introduced fully modified individuals (*ABCD*) in the centre of the range, except that the last column compares the wave speed for this case (light blue) to the case where only the fitness-valley component (*abCD*) is introduced. Other parameters: *δ* = 0.9 (drive rate), *s*_*d*_ = 0.02 (drive load), *s*_*t*_ = 0.9 (toxin load), *R* = *r* = 0.5 (recombination rate), D=0.2 (diffusion rate), and *L* = 200 (length of the spatial domain). Time and space were subdivided into ten steps each to approximate continuous time and space (i.e., using a spatial step size of 0.1).(EPS)

S10 Fig Spread of introduced genotypes across one-dimensional continuous space that is heterogeneous. The first three panels represent alleles frequencies along a one-dimensional spatial axis at times **(A)** *t* = 0, **(B)** *t* = 300, and **(C)** *t* = 600. The last panel **(D)** describes the speed of the cargo wave, measuring the frequency of either *C* or *D*, i.e. the speed of the pink curve in the first three graphs. The domain is heterogeneous, with steps of size 1 (middle half) and steps of size 2 (outer half). Other parameters as in S9 Fig.
(EPS)

S11 FigThe asymptotic wave speed for daisy quorum drive as a function of the spatial step size for different values of the payload cost *s*_*p*_ across a two-dimensional area.The three panels above the graph show the convex hull containing 80% of the population for either the *C* or *D* allele at *t* = 0 (dark blue contour), *t* = 100, *t* = 200 and *t* = 300 (light blue), with *s*_*p*_ = 0.1. Parameters are as follows: *R* = *r* = 0.5 (recombination rate), *s*_*d*_ = 0.02 (drive load), *s*_*t*_ = 0.9 (toxin load), *δ* = 0.9 (drive rate), *T* = 4000 (final time), *L* = 800 (length of the spatial domain), D=0.2 (diffusion rate), with each generation split into ten time steps to mimic continuous time and the spatial domain split into a series of patches (from 8000^2^ down to 267^2^, across the two dimensions) at increasing distances apart (from 0.1 to 3 spatial units).(EPS)

S1 Table Mating table for loci A and B. Table illustrates the gametes that come together to make a diploid individual (first two columns), their fitness (third column), frequency at birth (fourth column), and gametes produced (last four columns).
(PDF)

S2 Table Mating table for locus C and D. Table illustrates the gametes that come together to make a diploid individual (first two columns), their fitness (third column), frequency at birth (fourth column), and gametes produced (last four columns).
(PDF)

S3 Table Fitnesses when expression of the payload (*s*_*p*_) is multiplicative within and between loci. (PDF)

S4 Table Fitnesses when expression of the payload (*s*_*p*_) is recessive at locus C and at locus D, acting independently on each. (PDF)

S5 Table Fitnesses for a fitness valley created by swapping two haploinsufficient genes at alleles *C* and *D*, which also carry a dominant payload. (PDF)

S6 Table Relative fitness values used by Dhole et al. [8] and Champer et al. [9]. In those studies, the toxin load was set to *s*_*t*_ = 1.
(PDF)

## References

[1] National Academies of Sciences, Engineering, and Medicine. Gene drives on the horizon: advancing science, navigating uncertainty and aligning research with public values. National Academies Press; 2016. 27536751

[2] Wedell N, Price TAR, Lindholm AK. Gene drive: progress and prospects. Proceedings of the Royal Society B: Biological Sciences. 2019;286(1917):20192709. doi: 10.1098/rspb.2019.2709 31847764 PMC6939923

[3] Isaacs AT, Jasinskiene N, Tretiakov M, Thiery I, Zettor A, Bourgouin C, et al. Transgenic *Anopheles stephensi* coexpressing single-chain antibodies resist *Plasmodium falciparum* development. Proceedings of the National Academy of Sciences. 2012;109(28):E1922–E1930. doi: 10.1073/pnas.1207738109 22689959 PMC3396534

[4] Noble C, Min J, Olejarz J, Buchthal J, Chavez A, Smidler AL, et al. Daisy-chain gene drives for the alteration of local populations. Proceedings of the National Academy of Sciences. 2019;116(17):8275–8282. doi: 10.1073/pnas.1716358116 30940750 PMC6486765

[5] Turelli M, Barton NH. Why did the *Wolbachia* transinfection cross the road? drift, deterministic dynamics, and disease control. Evolution Letters. 2022;6(1):92–105. doi: 10.1002/evl3.270 35127140 PMC8802242

[6] Davis S, Bax N, Grewe P. Engineered underdominance allows efficient and economical introgression of traits into pest populations. Journal of Theoretical Biology. 2001;212(1):83–98. doi: 10.1006/jtbi.2001.2357 11527447

[7] Min J, Noble C, Najjar D, Esvelt K. Daisy quorum drives for the genetic restoration of wild populations. BioRxiv. 2017; p. 115618.

[8] Dhole S, Vella MR, Lloyd AL, Gould F. Invasion and migration of spatially self-limiting gene drives: A comparative analysis. Evolutionary Applications. 2018;11(5):794–808. doi: 10.1111/eva.12583 29875820 PMC5978947

[9] Champer J, Zhao J, Champer SE, Liu J, Messer PW. Population dynamics of underdominance Gene Drive Systems in continuous space. ACS Synthetic Biology. 2020;9(4):779–792. doi: 10.1021/acssynbio.9b00452 32142612 PMC8284921

[10] Burt A. Site-specific selfish genes as tools for the control and genetic engineering of natural populations. Proceedings of the Royal Society of London Series B: Biological Sciences. 2003;270(1518):921–928. doi: 10.1098/rspb.2002.2319 12803906 PMC1691325

[11] Sinkins SP, Gould F. Gene drive systems for insect disease vectors. Nature Reviews Genetics. 2006;7(6):427–435. doi: 10.1038/nrg1870 16682981

[12] Kyrou K, Hammond AM, Galizi R, Kranjc N, Burt A, Beaghton AK, et al. A CRISPR–Cas9 gene drive targeting doublesex causes complete population suppression in caged *Anopheles gambiae* mosquitoes. Nature Biotechnology. 2018;36(11):1062. doi: 10.1038/nbt.4245 30247490 PMC6871539

[13] Hammond A, Pollegioni P, Persampieri T, North A, Minuz R, Trusso A, et al. Gene-drive suppression of mosquito populations in large cages as a bridge between lab and field. Nature Communications. 2021;12(1):4589. doi: 10.1038/s41467-021-24790-6 34321476 PMC8319305

[14] Grunwald HA, Gantz VM, Poplawski G, Xu XRS, Bier E, Cooper KL. Super-Mendelian inheritance mediated by CRISPR-Cas9 in the female mouse germline. Nature. 2019;566(7742):105–109. doi: 10.1038/s41586-019-0875-2 30675057 PMC6367021

[15] DiCarlo JE, Chavez A, Dietz SL, Esvelt KM, Church GM. Safeguarding CRISPR-Cas9 gene drives in yeast. Nature Biotechnology. 2015;33(12):1250. doi: 10.1038/nbt.3412 26571100 PMC4675690

[16] Yang E, Metzloff M, Langmüller AM, Xu X, Clark AG, Messer PW, et al. A homing suppression gene drive with multiplexed gRNAs maintains high drive conversion efficiency and avoids functional resistance alleles. G3 Genes|Genomes|Genetics. 2022;12(6):jkac081. doi: 10.1093/g3journal/jkac081 35394026 PMC9157102

[17] Fuchs S, Garrood WT, Beber A, Hammond A, Galizi R, Gribble M, et al. Resistance to a CRISPR-based gene drive at an evolutionarily conserved site is revealed by mimicking genotype fixation. PLOS Genetics. 2021;17(10):e1009740. doi: 10.1371/journal.pgen.1009740 34610011 PMC8519452

[18] Champer J, Reeves R, Oh SY, Liu C, Liu J, Clark AG, et al. Novel CRISPR/Cas9 gene drive constructs reveal insights into mechanisms of resistance allele formation and drive efficiency in genetically diverse populations. PLoS genetics. 2017;13(7):e1006796. doi: 10.1371/journal.pgen.1006796 28727785 PMC5518997

[19] Unckless RL, Clark AG, Messer PW. Evolution of resistance against CRISPR/Cas9 gene drive. Genetics. 2017;205(2):827–841. doi: 10.1534/genetics.116.197285 27941126 PMC5289854

[20] Peach DA, Gries G. Mosquito phytophagy–sources exploited, ecological function, and evolutionary transition to haematophagy. Entomologia Experimentalis et Applicata. 2020;168(2):120–136. doi: 10.1111/eea.12852

[21] Kraus JM, Vonesh JR. Fluxes of terrestrial and aquatic carbon by emergent mosquitoes: a test of controls and implications for cross-ecosystem linkages. Oecologia. 2012;170:1111–1122. doi: 10.1007/s00442-012-2369-x 22707036

[22] Alphey N, Bonsall MB. Interplay of population genetics and dynamics in the genetic control of mosquitoes. Journal of The Royal Society Interface. 2014;11(93):20131071. doi: 10.1098/rsif.2013.1071 24522781 PMC3928937

[23] Deredec A, Burt A, Godfray HCJ. The population genetics of using homing endonuclease genes in vector and pest management. Genetics. 2008;179(4):2013–2026. doi: 10.1534/genetics.108.089037 18660532 PMC2516076

[24] Noble C, Adlam B, Church GM, Esvelt KM, Nowak MA. Current CRISPR gene drive systems are likely to be highly invasive in wild populations. eLife. 2018;7:e33423. doi: 10.7554/eLife.33423 29916367 PMC6014726

[25] Rode NO, Estoup A, Bourguet D, Courtier-Orgogozo V, Débarre F. Population management using gene drive: molecular design, models of spread dynamics and assessment of ecological risks. Conservation Genetics. 2019;20:671–690. doi: 10.1007/s10592-019-01165-5

[26] Long KC, Alphey L, Annas GJ, Bloss CS, Campbell KJ, Champer J, et al. Core commitments for field trials of gene drive organisms. Science. 2020;370(6523):1417–1419. doi: 10.1126/science.abd1908 33335055

[27] Akbari OS, Bellen HJ, Bier E, Bullock SL, Burt A, Church GM, et al. Safeguarding gene drive experiments in the laboratory. Science. 2015;349(6251):927–929. doi: 10.1126/science.aac7932 26229113 PMC4692367

[28] Adelman Z, Akbari O, Bauer J, Bier E, Bloss C, Carter SR, et al. Rules of the road for insect gene drive research and testing. Nature Biotechnology. 2017;35(8):716–718. doi: 10.1038/nbt.3926 28787415 PMC5831321

[29] Li M, Yang T, Kandul NP, Bui M, Gamez S, Raban R, et al. Development of a confinable gene drive system in the human disease vector *Aedes aegypti*. eLife. 2020;9:e51701. doi: 10.7554/eLife.51701 31960794 PMC6974361

[30] Min J, Noble C, Najjar D, Esvelt KM. Daisyfield gene drive systems harness repeated genomic elements as a generational clock to limit spread. BioRxiv. 2017; p. 104877.

[31] Gantz VM, Bier E. The dawn of active genetics. Bioessays. 2016;38(1):50–63. doi: 10.1002/bies.201500102 26660392 PMC5819344

[32] Wu B, Luo L, Gao XJ. Cas9-triggered chain ablation of *cas9* as a gene drive brake. Nature Biotechnology. 2016;34(2):137. doi: 10.1038/nbt.3444 26849513 PMC5326742

[33] Taxiarchi C, Beaghton A, Don NI, Kyrou K, Gribble M, Shittu D, et al. A genetically encoded anti-CRISPR protein constrains gene drive spread and prevents population suppression. Nature Communications. 2021;12(1):3977. doi: 10.1038/s41467-021-24214-5 34172748 PMC8233359

[34] Verkuijl SA, Anderson MAE, Alphey L, Bonsall MB. Daisy-chain gene drives: The role of low cut-rate, resistance mutations, and maternal deposition. PLoS Genetics. 2022;18(9):e1010370. doi: 10.1371/journal.pgen.1010370 36121880 PMC9521892

[35] Serebrovsky AS. On the possibility of a new method for the control of insect pests. In: Sterile-Male Technique for Eradication or Control of Harmful Insects. Proceedings of a Panel on Application of the Sterile-Male Technique for the Eradication or Control of Harmful Species of Insects. Vienna: Division of Atomic Energy in Food and Agriculture; 1968. p. 123–237.

[36] Curtis CF. Possible use of translocations to fix desirable genes in insect pest populations. Nature. 1968;218(5139):368–369. doi: 10.1038/218368a0 5649682

[37] Gould F, Huang Y, Legros M, Lloyd AL. A Killer–Rescue system for self-limiting gene drive of anti-pathogen constructs. Proceedings of the Royal Society B: Biological Sciences. 2008;275(1653):2823–2829. doi: 10.1098/rspb.2008.0846 18765342 PMC2572679

[38] Reeves RG, Bryk J, Altrock PM, Denton JA, Reed FA. First steps towards underdominant genetic transformation of insect populations. PLOS ONE. 2014;9(5):e97557. doi: 10.1371/journal.pone.0097557 24844466 PMC4028297

[39] Akbari OS, Matzen KD, Marshall JM, Huang H, Ward CM, Hay BA. A synthetic gene drive system for local, reversible modification and suppression of insect populations. Current Biology. 2013;23(8):671–677. doi: 10.1016/j.cub.2013.02.059 23541732 PMC8459379

[40] Altrock PM, Traulsen A, Reeves RG, Reed FA. Using underdominance to bi-stably transform local populations. Journal of Theoretical Biology. 2010;267(1):62–75. doi: 10.1016/j.jtbi.2010.08.004 20691703

[41] Altrock PM, Traulsen A, Reed FA. Stability properties of underdominance in finite subdivided populations. PLoS computational biology. 2011;7(11):e1002260. doi: 10.1371/journal.pcbi.1002260 22072956 PMC3207953

[42] Marshall JM, Hay BA. Confinement of gene drive systems to local populations: A comparative analysis. Journal of Theoretical Biology. 2012;294:153–171. doi: 10.1016/j.jtbi.2011.10.032 22094363 PMC3260013

[43] Edgington MP, Alphey LS. Population dynamics of engineered underdominance and Killer-Rescue gene drives in the control of disease vectors. PLOS Computational Biology. 2018;14(3):e1006059. doi: 10.1371/journal.pcbi.1006059 29570717 PMC5884568

[44] Barton NH. The dynamics of hybrid zones. Heredity. 1979;43(3):341–359. doi: 10.1038/hdy.1979.87

[45] Barton NH, Turelli M. Spatial waves of advance with bistable dynamics: cytoplasmic and genetic analogues of Allee effects. The American Naturalist. 2011;178(3):E48–E75. doi: 10.1086/661246 21828986

[46] Connolly JB, Mumford JD, Fuchs S, Turner G, Beech C, North AR, et al. Systematic identification of plausible pathways to potential harm via problem formulation for investigational releases of a population suppression gene drive to control the human malaria vector *Anopheles gambiae* in West Africa. Malaria Journal. 2021;20(1):170. doi: 10.1186/s12936-021-03674-6 33781254 PMC8006393

[47] Li J, Champer J. Harnessing *Wolbachia* cytoplasmic incompatibility alleles for confined gene drive: A modeling study. PLoS Genetics. 2023;19(1):e1010591. doi: 10.1371/journal.pgen.1010591 36689491 PMC9894560

[48] Edgington MP, Harvey-Samuel T, Alphey L. Split drive killer-rescue provides a novel threshold-dependent gene drive. Scientific Reports. 2020;10(1):20520. doi: 10.1038/s41598-020-77544-7 33239631 PMC7689494

[49] Faber NR, McFarlane GR, Gaynor RC, Pocrnic I, Whitelaw CBA, Gorjanc G. Novel combination of CRISPR-based gene drives eliminates resistance and localises Spread. Scientific Reports. 2021;11(1):3719. doi: 10.1038/s41598-021-83239-4 33664305 PMC7933345

[50] Terradas G, Buchman AB, Bennett JB, Shriner I, Marshall JM, Akbari OS, et al. Inherently confinable split-drive systems in *Drosophila*. Nature Communications. 2021;12(1):1480. doi: 10.1038/s41467-021-21771-7 33674604 PMC7935863

[51] Dhole S, Lloyd AL, Gould F. Tethered homing gene drives: a new design for spatially restricted population replacement and suppression. Evolutionary applications. 2019;12(8):1688–1702. doi: 10.1111/eva.12827 31462923 PMC6708424

[52] Geci R, Willis K, Burt A. Gene drive designs for efficient and localisable population suppression using Y-linked editors. PLoS Genetics. 2022;18(12):e1010550. doi: 10.1371/journal.pgen.1010550 36574454 PMC9829173

[53] Champer J, Champer SE, Kim IK, Clark AG, Messer PW. Design and analysis of CRISPR-based underdominance toxin-antidote gene drives. Evolutionary Applications. 2020;14(4):1052–1069. doi: 10.1111/eva.13180 33897820 PMC8061266

[54] Girardin L, Débarre F. Demographic feedbacks can hamper the spatial spread of a gene drive. Journal of Mathematical Biology. 2021;83(6):67. doi: 10.1007/s00285-021-01702-2 34862932

[55] Kläy L, Girardin L, Calvez V, Débarre F. Pulled, pushed or failed: the demographic impact of a gene drive can change the nature of its spatial spread. Journal of Mathematical Biology. 2023;87(2):30. doi: 10.1007/s00285-023-01926-4 37454310

