# Supplementary material for: Modelling daisy quorum drive: A short-term bridge across engineered fitness valleys
Source: PLoS Genet. 2024 May 16;20(5):e1011262. doi: 10.1371/journal.pgen.1011262 (PMC11135765; doi:10.1371/journal.pgen.1011262)
Supplement: S1 Appendix — (PDF) [file pgen.1011262.s001.pdf]

Modelling daisy quorum drive: a short-term bridge across engineered fitness valleys (PLoS Genetics 2024)

Frederik JH de Haas, & Léna Kläy, Florence Débarre, Sarah P Otto*
* otto@zoology.ubc.ca

S1 Appendix. Daisy quorum drive in a single population

S1.1 Daisy-chain length

The length of the daisy chain is an important determinant of the maximum attainable frequency of the payload. Here we illustrate this effect for daisy chains of different lengths by tracking the maximum frequency attained by the last element in the chain. As the number of loci involved in the daisy chain increases, there is a stronger and longer force pushing the last drive element to a higher frequency (S1 Fig). The inset panel shows an example of the dynamics of a daisy-chain system with three drive loci, starting at a frequency of 0.02 (star) and attaining a maximum frequency of 0.52 (square).

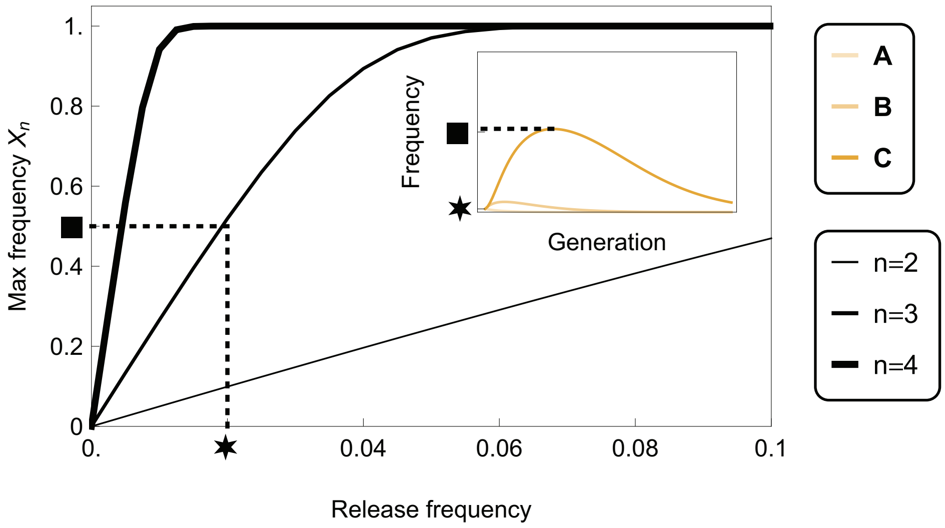

S1 Fig. Maximum frequency of the last element in an n length daisy-chain construct in a single population. A daisy chain of size n is designed by repeating locus **A** in Fig 2 for $\mathbf{A}_1$,..., $\mathbf{A}_{n-1}$, each carrying a gRNA targeting the next locus in the chain. All elements of the daisy-chain carry a drive load $s_d = 0.05$. The frequency of the nth transgenic allele is denoted by X_n. Other parameters are $\delta = 1$, $R = 0.5$.

S1.2 Mating and gamete production

Here we enumerate the various possible matings and gametes produced for the 4-locus daisy quorum system explored in the main text (Fig 2). S1 Table gives the mating table for the dynamics at loci **A** and **B** (the daisy-chain component). S2 Table provides the mating table for the dynamics at loci **C** and **D** (the fitness-valley component).

Gamete 1	Gamete 2	Fitness	Freq	Gametes produced			
				ab	aB	Ab	AB
ab	ab	1	X_{ab}^2	1			
ab	aB	$(1-s_d)$	$2X_{ab}X_{aB}$	$\frac{1}{2}$	$\frac{1}{2}$		
ab	Ab	$(1-s_d)$	$2X_{ab}X_{Ab}$	$\frac{1}{2}$		$\frac{1}{2}$	
ab	AB	$(1-s_d)^2$	$2X_{ab}X_{AB}$	$\frac{1}{2}(1-\delta)(1-r)$	$\frac{1}{2}(\delta+(1-\delta)r)$	$\frac{1}{2}(1-\delta)r$	$\frac{1}{2}(1-(1-\delta)r)$
aB	aB	$(1-s_d)^2$	X_{aB}^2		1		
aB	Ab	$(1-s_d)^2$	$2X_{aB}X_{Ab}$	$\frac{1}{2}(1-\delta)r$	$\frac{1}{2}(1-(1-\delta)r)$	$\frac{1}{2}(1-\delta)(1-r)$	$\frac{1}{2}(\delta+(1-\delta)r)$
aB	AB	$(1-s_d)^3$	$2X_{aB}X_{AB}$		$\frac{1}{2}$	$\frac{1}{2}$	
Ab	Ab	$(1-s_d)^2$	X_{Ab}^2			1	
Ab	AB	$(1-s_d)^3$	$2X_{Ab}X_{AB}$			$\frac{1}{2}(1-\delta)$	$\frac{1}{2}(1+\delta)$
AB	AB	$(1-s_d)^4$	X_{AB}^2				1

S1 Table. Mating table for loci A and B. Table illustrates the gametes that come together to make a diploid individual (first two columns), their fitness (third column), frequency at birth (fourth column), and gametes produced (last four columns).

Gamete 1	Gamete 2	Fitness	Freq	Gametes produced			
				cd	cD	Cd	CD
cd	cd	1	X_{cd}^2	1			
cd	cD	$(1-s_t)(1-s_p)$	$2X_{cd}X_{cD}$	$\frac{1}{2}$	$\frac{1}{2}$		
cd	Cd	$(1-s_t)(1-s_p)$	$2X_{cd}X_{Cd}$	$\frac{1}{2}$		$\frac{1}{2}$	
cd	CD	$(1-s_p)$	$2X_{cd}X_{CD}$	$\frac{1}{2}(1-r)$	$\frac{1}{2}r$	$\frac{1}{2}r$	$\frac{1}{2}(1-r)$
cD	cD	$(1-s_t)(1-s_p)$	X_{cD}^2		1		
cD	Cd	$(1-s_p)$	$2X_{cD}X_{Cd}$	$\frac{1}{2}r$	$\frac{1}{2}(1-r)$	$\frac{1}{2}(1-r)$	$\frac{1}{2}r$
cD	CD	$(1-s_p)$	$2X_{cD}X_{CD}$		$\frac{1}{2}$	$\frac{1}{2}$	
Cd	Cd	$(1-s_t)(1-s_p)$	X_{Cd}^2			1	
Cd	CD	$(1-s_p)$	$2X_{Cd}X_{CD}$			$\frac{1}{2}$	$\frac{1}{2}$
CD	CD	$(1-s_p)$	X_{CD}^2				1

S2 Table. Mating table for locus C and D. Table illustrates the gametes that come together to make a diploid individual (first two columns), their fitness (third column), frequency at birth (fourth column), and gametes produced (last four columns).

S1.3 Threshold frequencies with a dominant payload

Without drive, the fitness valley created by the toxin-antidote construct at loci **C** and **D** can be crossed if the construct begins at a high enough frequency. S2 Fig illustrates the location of the stable equilibria (blue and grey vertices) and the separatrix (the boundary separating two basins of attraction in a dynamical model, calculated numerically) for different payloads (s_p) when the toxin creating the fitness valley is relatively weak (panel **(A)**: $s_t = 0.1$) or very strong (panel **(B)**: $s_t = 1.0$). The special case of a lethal toxin load ($s_t = 1.0$) and no payload ($s_p = 0$) corresponds to the case considered by Davis et al. [6] and matches the yellow curve in S2 Fig panel B (see their Fig 6). Example dynamics confirm that the system converges to either fixation on the wildtype (cd) or fitness-valley construct (CD) depending on whether initialized to the left or right side of the separatrix.

As the cost of the payload increases (larger s_p), the separatrix moves to the right in S2 Fig, reducing the basin of attraction of the engineered fitness-valley construct (haplotype CD). Higher payloads thus make it harder to drive such constructs into a population, but they also provide stronger fitness effects when the construct is fixed. By contrast, as the toxin load increases (larger s_t), the separatrix moves left, and the basin of attraction to CD increases. While somewhat counterintuitive, this occurs because the toxin load can be suppressed by even one copy of the alternate allele, making haplotypes Cd and cD strongly selected against when cd is common (the wildtype) but only weakly selected against when CD is common, causing the fitness surface to fall faster near cd than near CD.

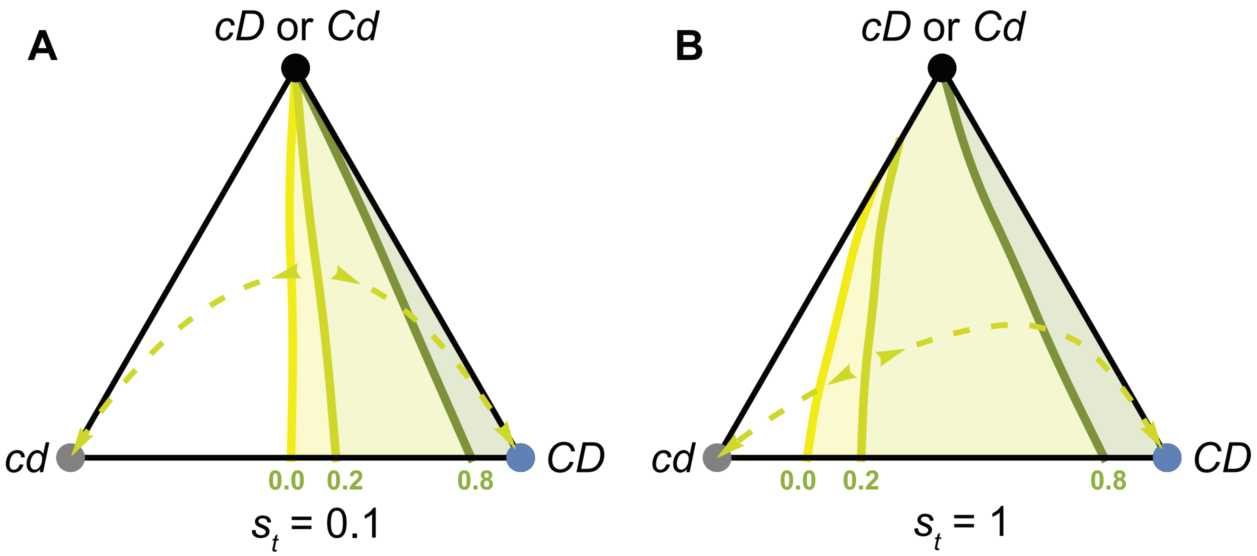

S2 Fig. Ternary plot illustrating the separatrix (the boundary separating basins of attraction to different fixed points) for the two-locus fitness-valley construct. The construct creates a toxin load of $s_t = 0.1$ in panel **(A)** or $s_t = 1.0$ in panel **(B)** and a payload of $s_p = 0.0, 0.2$ and 0.8 (green curves). Locus **C** and **D** recombine freely ($r = \frac{1}{2}$). The dashed curves are example dynamics for $s_p = 0.2$.

S1.4 Full dynamics

By assuming a constant initial drive force δ_c, the invasion analysis in the main text (Eq 4) ignores changes to the genetic associations between the daisy-chain and fitness-valley components. Here we compare the predictions from this invasion analysis to full numerical analyses of the four-locus daisy quorum system (Fig 2).

S3 Fig illustrates the dynamics following the introduction of the $ABCD$ construct at frequency f_0 with either a low toxin load ($s_t = 0.1$, panels **(A)**,**(B)**) or a high toxin load ($s_t = 0.9$, panels **(C)**,**(D)**). Drive is initially strong enough ($\delta = 0.9$) that the toxin-antidote alleles C and D spread when rare (from equation 5: $\delta_c^* = 0.23$ in panels A,B and 0.49 in panels **(C)**,**(D)**).

Over time, recombination breaks down the strong initial genetic association between the driver (B) and driven alleles (C or D). Because drive is only relevant in Cc or Dd heterozygotes, we measure the drive phenotype as the fraction of these heterozygotes that experience drive (with genotype Bb or BB). The drive phenotype (solid orange curve, giving $X_{B|Cc}^2 + 2X_{B|Cc}X_{b|Cc}$) starts at one when the alleles are introduced together in the $ABCD$ construct but drops over time. If the fitness-valley construct starts at too low a frequency ($f_0 = 0.015$, left panels) and is not driven to a high enough frequency before drive is exhausted, then the fitness-valley construct is lost. Starting at a slightly higher initial frequency ($f_0 = 0.02$, right panels), the frequency of allele C (or equivalently D; blue curve) crosses over the fitness valley and enters the basin of attraction of the fixation equilibrium where $X_C = X_D = 1$. In the terminology of Barton [44] exploring one-locus underdominance, the fitness-valley construct initially rides a "Fisherian" wave, rising from low initial frequency due to gene drive, but it then completes the transition to fixation by riding a "bistable" wave, as long as it passes the unstable equilibrium before drive is exhausted.

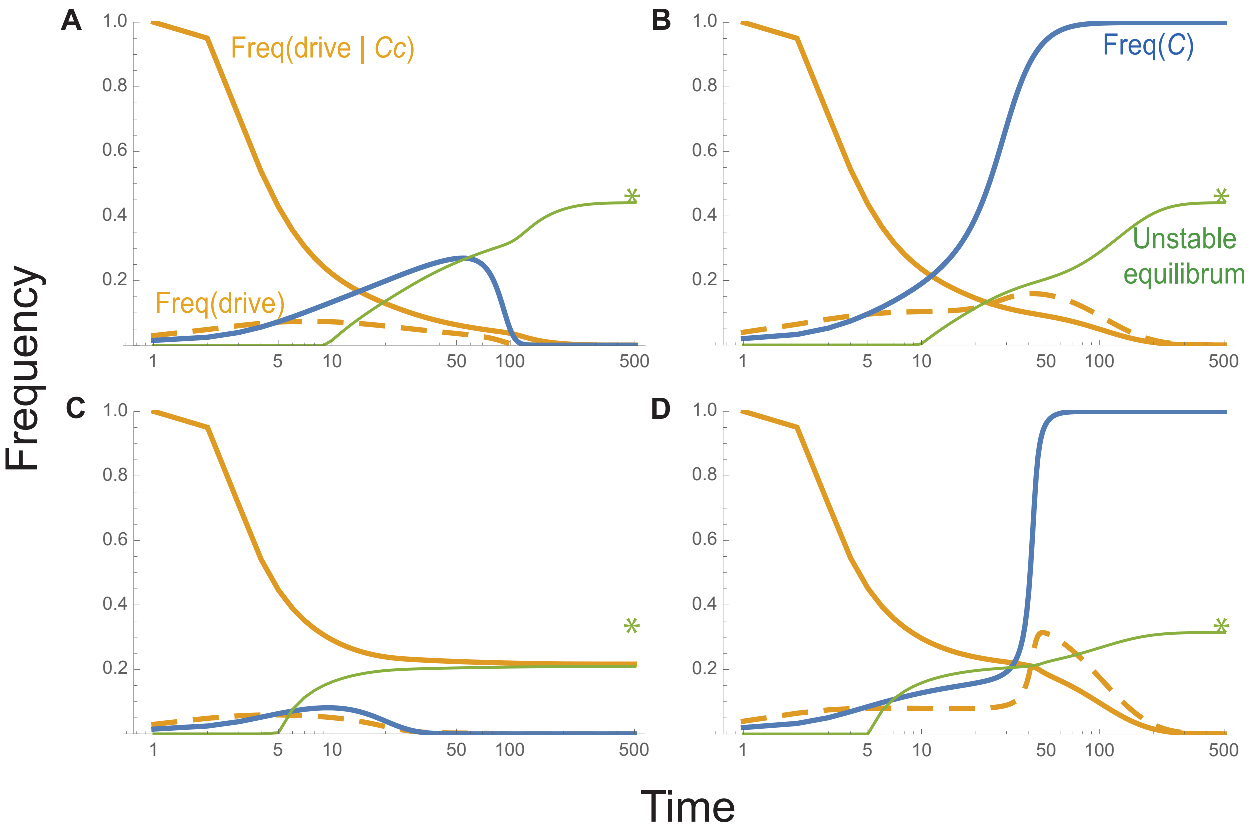

S3 Fig. The dynamics of the full four-locus daisy quorum drive. The toxin load is low in panels **(A)**,**(B)** ($s_t = 0.1$) and high in panels **(C)**,**(D)** ($s_t = 0.9$). The frequency of the toxic allele C (equivalently D) is shown in blue, with $ABCD$ introduced at frequency $f_0 = 0.015$ (panels **(A)**,**(C)**) or $f_0 = 0.02$ (panels **(B)**,**(D)**). The toxin-antidote alleles C and D together rise to fixation (crossing the fitness valley) only when started at a high enough initial frequency to suppress each other's toxicity (panels **(B)**,**(D)**). The frequency of the drive phenotype (including both BB and Bb genotypes) is shown among Cc heterozygotes (equivalently Dd heterozygotes; solid orange) and in the full population (dashed orange). The green curve shows the position of the unstable equilibrium of the fitness-valley construct, given the current frequency of the drive phenotype in Cc (or in Dd) heterozygotes, with the star indicating the position once drive has disappeared. Parameters: $\delta = 0.9$, $R = r = 0.5$, $s_d = 0.02$, $s_p = 0.1$.

To guide the eye, the green curve gives the position of the unstable equilibrium for the two-locus fitness-valley construct **CD** if the force of drive were fixed at its current value among Cc or Dd heterozgyotes (measuring the force of drive as $X^2_{B|Cc} + 2X_{B|Cc}X_{b|Cc}$, given by the orange solid curve, times δ). While this is not precisely the separatrix in the full four-locus system because genetic associations between the daisy chain component and the fitness-valley component are ignored, we can see that the fitness-valley construct (blue curve) must be introduced at a high enough frequency that it is pushed above the unstable equilibrium in the two-locus system, given the current force of drive (green curve), before the driver allele B has disappeared from the population (orange dashed curve).

S1.5 Multiplicative payload

In the main text we focus on fitnesses with a dominant payload (Table 1), in which case fixation of the CD haplotype is locally stable. If we assume different payload regimes (e.g., multiplicative or recessive effects on individual fitness), then fixation of the CD haplotype is no longer a stable equilibrium, as described below.

S3 Table gives the fitnesses for each genotype at loci **C** and **D** assuming a multiplicative payload. The governing equations for the fitness-valley component are then given by equations 2 using the entries of S3 Table.

	cd	cD	Cd	CD
cd	1	$(1-s_t)(1-s_p)^{1/4}$	$(1-s_t)(1-s_p)^{1/4}$	$(1-s_p)^{1/2}$
cD	$(1-s_t)(1-s_p)^{1/4}$	$(1-s_t)(1-s_p)^{1/2}$	$(1-s_p)^{1/2}$	$(1-s_p)^{3/4}$
Cd	$(1-s_t)(1-s_p)^{1/4}$	$(1-s_p)^{1/2}$	$(1-s_t)(1-s_p)^{1/2}$	$(1-s_p)^{3/4}$
CD	$(1-s_p)^{1/2}$	$(1-s_p)^{3/4}$	$(1-s_p)^{3/4}$	$1-s_p$

S3 Table. Fitnesses when expression of the payload (s_p) is multiplicative within and among loci.

Analysis of the equilibria of this fitness-valley system reveals that fixation of the wildtype alleles ($X_{cd} = 1$) is stable, but fixation of the fitness-valley construct is not ($X_{CD} = 1$). In addition, there are two internal equilibria, one of which is unstable (green) and one stable (blue), as illustrated in S4 Fig. The stable and unstable equilibrium move towards each other as the payload increases. This means that a drive requires a higher initial frequency to invade and will spread to a lower equilibrium frequency as the payload increases in strength. As in the dominant case considered in the main text, increasing the payload s_p or decreasing the toxin load s_t shifts the unstable equilibrium (green) further up and away from the wildtype corner (cd),

The same invasion analysis as in section 2.3 reveals that the necessary condition for drive to be strong enough for the transgenic alleles C and D to spread when introduced is:

$$\delta_c > \delta_c^* = \min[\frac{\frac{1}{\sqrt[4]{1-s_p}} - (1-s_t)}{1-s_t}, \frac{\frac{\sqrt{\sqrt{1-s_p}-(1-s_p)+r\left(2-2s_p-\sqrt{1-s_p}\right)}}{\sqrt{1-s_p}} - r}{1-r}] \quad (6)$$

For unlinked loci **C** and **D** ($r = 1/2$) and a multiplicative payload, the maximum payload that can be carried for a fitness-valley construct to increase initially in frequency is $s_p = 15/16$, which requires a maximal drive force $\delta_c = 1$ and a minimal toxin load $s_t \approx 0$.

S1.6 Recessive payload

S4 Table presents the relative fitness of individuals when the payload is recessive at each locus. We assume that the fitness-valley construct is built by disrupting alleles at two selectively important and haplosufficient genes, **C** and **D**, and that these genes act independently to determine fitness according to S4 Table. The governing equations for the fitness-valley component are then given by equations 2 using the entries of S4 Table.

In S5 Fig, we illustrate the location of the equilibria of this model for $s_t = 1.0$ and $s_t = 0.1$, where there are again two internal equilibrium (one unstable in green and one stable in blue). We see that similar to the analysis in the main text for a dominant payload, an

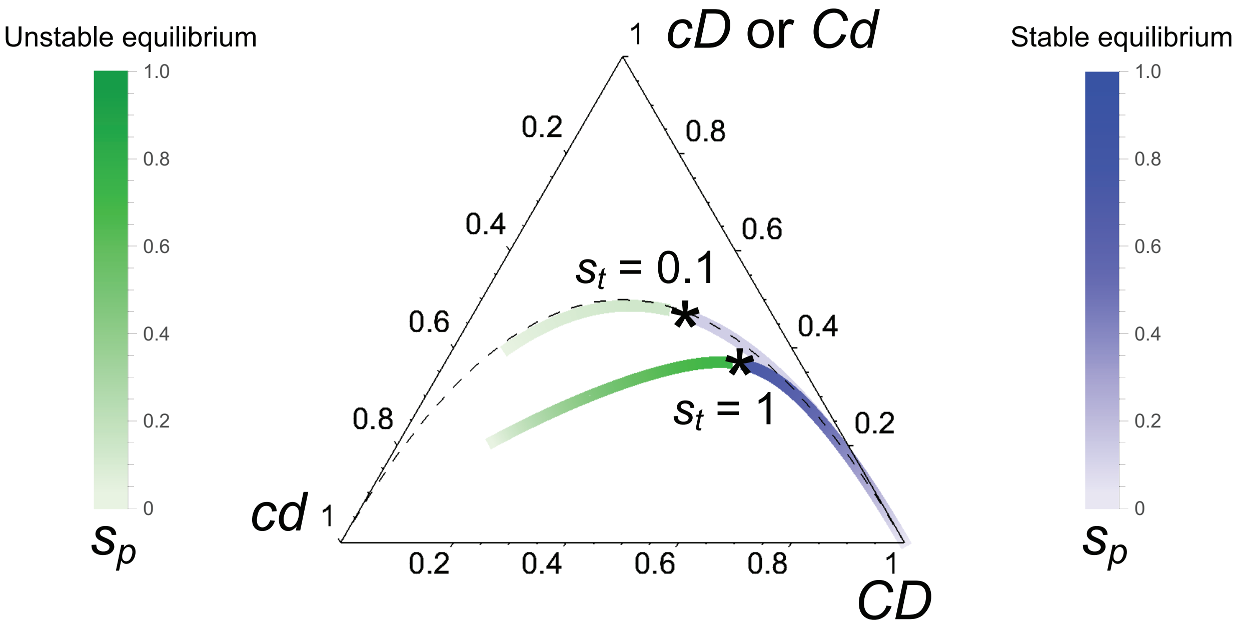

S4 Fig. Equilibria with multiplicative expression of the payload (S3 Table). The coloured curves indicate the internal unstable (green) and stable (blue) equilibria for different payload values s_p, either with a low toxin load ($s_t = 0.1$, top curves) or a high toxin load ($s_t = 1$, bottom curves). For a given payload, s_p, starting to the right of the green point will lead the system to approach the blue point with the same color saturation (s_p value). Recombination rate r is $\frac{1}{2}$, and the dashed curve indicates when there is no linkage disequilibrium between the two loci. If the payload is too strong relative to the toxin load, the internal equilibria become complex, as denoted by the $*$ (for $s_p \geq 0.11$ with $s_t = 0.1$ and for $s_p \geq 0.73$ with $s_t = 1$), at which point only the wildtype equilibrium is stable, and the fitness-valley construct is lost. Recombination rate r is $\frac{1}{2}$, and the dashed curve indicates when there is no linkage disequilibrium between the two loci.

	cd	*cD*	*Cd*	*CD*
cd	1	$1 - s_t$	$1 - s_t$	1
cD	$1 - s_t$	$(1 - s_t)(1 - s_p)^{1/2}$	1	$(1 - s_p)^{1/2}$
Cd	$1 - s_t$	1	$(1 - s_t)(1 - s_p)^{1/2}$	$(1 - s_p)^{1/2}$
CD	1	$(1 - s_p)^{1/2}$	$(1 - s_p)^{1/2}$	$1 - s_p$

S4 Table. Fitnesses when expression of the payload (s_p) is recessive at locus C and at locus D, acting independently on each.

increase in the payload or a reduction in the toxin load move the unstable equilibrium further up and to the right (S5 Fig). Consequently, a higher introduction frequency is required for alleles C and D to spread (further away from the wildtype cd haplotype on the bottom left).

An invasion analysis shows that a necessary condition for this construct to spread when first introduced is:

$$\delta_c > \delta_c^* = \min[\frac{s_t}{1 - s_t}, \frac{\sqrt{r}}{\sqrt{r} + 1}], \tag{7}$$

which is satisfied when the drive force δ_c is strong enough relative to the toxin load or recombination rate but which does not depend on the magnitude of a recessive payload.

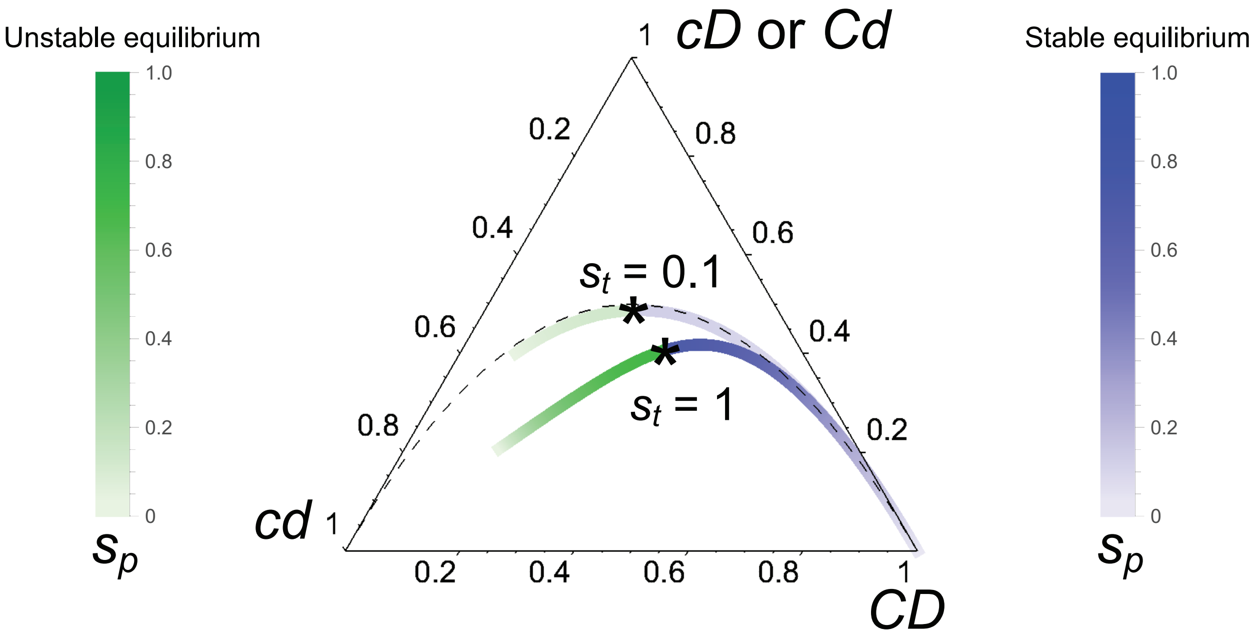

S5 Fig. Equilibria with recessive expression of the payload (S4 Table). The coloured curves indicate the internal unstable (green) and stable (blue) equilibria for different payload values s_p, either with a low toxin load ($s_t = 0.1$, top curves) or a high toxin load ($s_t = 1$, bottom curves). For a given payload, s_p, starting to the right of the green point will lead the system to approach the blue point with the same color saturation (s_p value). If the payload is too strong relative to the toxin load, the internal equilibria become complex, as denoted by the $*$ (for $s_p \geq 0.098$ with $s_t = 0.1$ and for $s_p \geq 0.71$ with $s_t = 1$), at which point only the wildtype equilibrium is stable, and the fitness-valley construct is lost. Recombination rate r is $\frac{1}{2}$, and the dashed curve indicates when there is no linkage disequilibrium between the two loci.

S1.7 Fitness valley via haploinsufficient gene swapping

When the fitness valley is created by swapping two haploinsufficient genes as proposed by Min et al. [7], individuals have reduced fitness unless they carry two copies of both genes **C** and **D**, either encoded by the original wildtype allele or the swapped allele at the other locus, as in S5 Table. Here we consider the general case by allowing partial loss in fitness by s_t when the functions of the haploinsufficient genes are disrupted, where $s_t = 1$ if disruption is lethal as considered by [7]. As detailed in the associated *Mathematica* file archived on Zenodo (doi:10.5281/zenodo.10904198), results for fitness-valley constructs created by swapping haploinsufficient genes are similar to those using a toxin-antidote system. Specifically, invasion criteria are unchanged (Eqs 4 and 5 and Fig 3A). The most important difference is that the threshold for spread of CD (the separatrix) is always above 50% (unlike S2 Fig). This is because the fitness valley created by haploinsufficient gene swapping is symmetric (two alleles are needed to carry out both gene functions), whereas the toxin-antidote system is asymmetric (only one copy of the antidote is sufficient to neutralize the toxin). Numerical results of the full four-locus daisy quorum drive indicate that stronger drive is thus needed to fix the construct CD (compare S6 Fig to 4).

	cd	cD	Cd	CD
cd	1	$(1-s_t)(1-s_p)$	$(1-s_t)(1-s_p)$	$(1-s_p)$
cD	$(1-s_t)(1-s_p)$	$(1-s_t)(1-s_p)$	$(1-s_p)$	$(1-s_t)(1-s_p)$
Cd	$(1-s_t)(1-s_p)$	$(1-s_p)$	$(1-s_t)(1-s_p)$	$(1-s_t)(1-s_p)$
CD	$(1-s_p)$	$(1-s_t)(1-s_p)$	$(1-s_t)(1-s_p)$	$(1-s_p)$

S5 Table. Fitnesses for a fitness valley created by swapping two haploinsufficient genes at alleles C and D, which also carry a dominant payload.

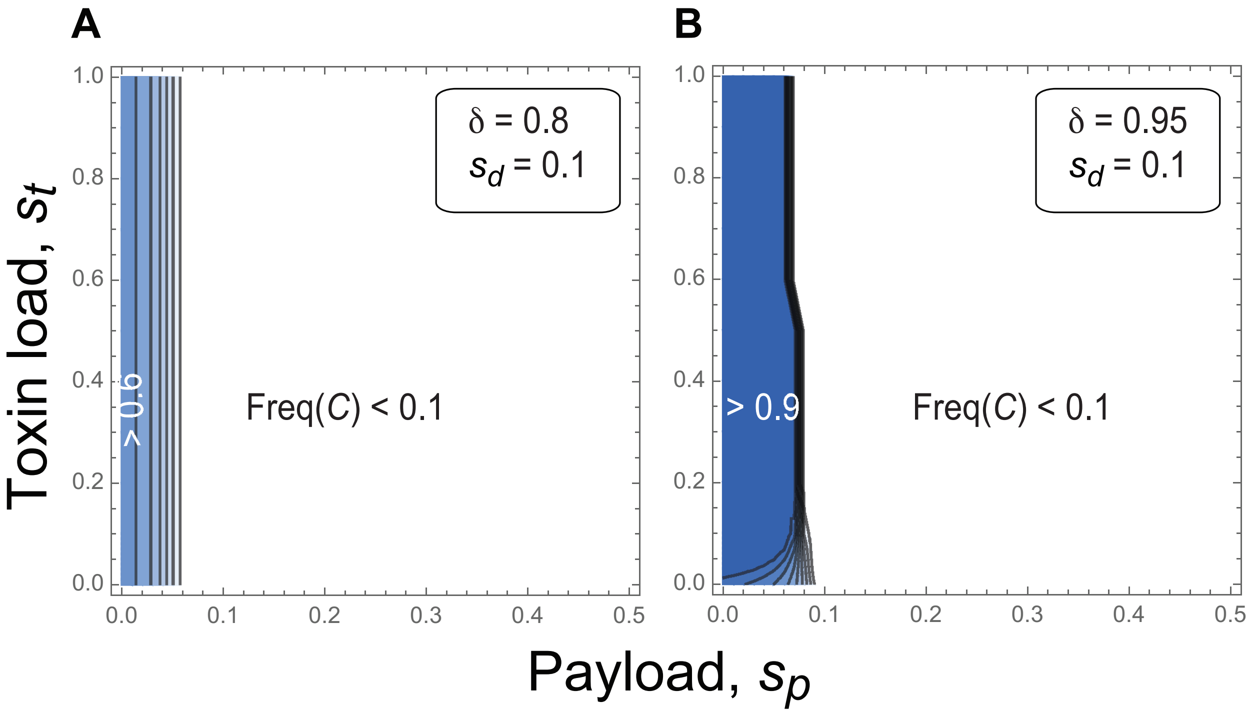

S6 Fig. Parameters allowing fitness-valley crossing in the four-locus model of daisy quorum drive when C and D carry swapped versions of two haploinsufficient genes as in [7]. The frequency of the fitness-valley construct C (or equivalently D) at generation $t = 100$ is illustrated across all values of the toxin load and payload where initial spread is possible. Only in the darker regions (constructs above 50%) does the construct remain at $t = 500$. Only in the darker regions (constructs above 50%) does the construct remain at $t = 500$. Panel **(A)**: weaker drive with $\delta = 0.8$; panel **(B)**: stronger drive with $\delta = 0.95$. Other parameters are: $f_0 = 0.05$, $s_d = 0.1$, and $R = r = 0.5$.

S1.8 Asymmetrical and multiplicative payload

Dhole et al. [8] and Champer et al. [9] studied another variant of the two-locus two-toxin-antidote system in which the toxin is lethal ($s_t = 1$) and the payload is carried by only one of introduced alleles (C in S6 Table), with multiplicative fitness effects ($1 - s_p$ for CC homozygotes and $\sqrt{1 - s_p}$ for Cc heterozygotes). The drive allele at the other locus (D in S6 Table) carries no payload, only a toxin and an antidote to the toxin carried by allele C.

	cd	cD	Cd	CD
cd	1	$(1-s_t)$	$(1-s_t)(1-s_p)^{1/2}$	$(1-s_p)^{1/2}$
cD	$(1-s_t)$	$(1-s_t)$	$(1-s_p)^{1/2}$	$(1-s_p)^{1/2}$
Cd	$(1-s_t)(1-s_p)^{1/2}$	$(1-s_p)^{1/2}$	$(1-s_t)(1-s_p)$	$(1-s_p)$
CD	$(1-s_p)^{1/2}$	$(1-s_p)^{1/2}$	$(1-s_p)$	$(1-s_p)$

S6 Table. Relative fitness values used by Dhole et al. [8] and Champer et al. [9]. In those studies, the toxin load was set to $s_t = 1$.
