# Supplementary material for: Modelling daisy quorum drive: A short-term bridge across engineered fitness valleys
Source: PLoS Genet. 2024 May 16;20(5):e1011262. doi: 10.1371/journal.pgen.1011262 (PMC11135765; doi:10.1371/journal.pgen.1011262)
Supplement: S2 Appendix — (PDF) [file pgen.1011262.s002.pdf]

Modelling daisy quorum drive: a short-term bridge across engineered fitness valleys (PLoS Genetics 2024)

Frederik JH de Haas, & Léna Kläy, Florence Débarre, Sarah P Otto*
* otto@zoology.ubc.ca

S2 Appendix. Individual-based simulations with discrete patches

Individual-based simulations in C^{++} were developed to explore whether our results hold for finite population sizes arrayed across multiple patches (code archived on Zenodo doi:10.5281/zenodo.10904198). We use the same fitness regime as in the main text (dominant payload expression). We implement the following life cycle in a single isolated population with carrying capacity K and then expand to consider migration among 101 patches in a linear stepping-stone array with the construct introduced into the central patch:

1. Calculate the expected frequency of genotypes predicted under the deterministic model.
2. Calculate mean fitness, $\overline{W}$, and draw the number of offspring in the next generation N^o_{t+1} as a Poisson random variate with rate parameter $\lambda = \overline{W} \times F \times N_t$. Here, F represents the average number of offspring per individual with wildtype fitness. Density dependence with a hard carrying capacity was then imposed, so that the total number of offspring in the next generation was set to $N_{t+1} = \min[N^o_{t+1}, K]$.
3. Genotypes were then drawn for the N_{t+1} offspring from a multinomial distribution with the deterministic offspring frequencies predicted based on the parental genotypes.

We repeat these steps for t_{max} generations and show results in S7 Fig and S8 Fig for $F = 1.2$ and $F = 1.05$, respectively. With $F = 1.2$, and a payload of $s_p = 0.1$, the average number of offspring per individual carrying the payload remains above unity ($0.9 \times 1.2 = 1.08$). However, the toxin load $s_t = 0.9$ causes a dip in population size (black dashed curve in S7 Fig) as the payload increases in frequency. Once the payload has fixed, the toxin load is no longer expressed, and the population recovers to its carrying capacity, regardless of the migration rate. For $F = 1.05$, however, the expected number of offspring per parent experiencing the payload falls below one ($0.9 \times 1.05 = 0.945$), meaning that the population is unable to sustain itself once the payload becomes common and declines to extinction in the absence of migration (top left panel in S8 Fig). With migration, however, population suppression is only transient. As the local population declines in size, proportionally more migrants enter the patch from neighboring sites. This proportionally rising inflow of wildtype individuals prevents fixation of the payload and eventually helps the population recover to its carrying capacity with only wildtype individuals.

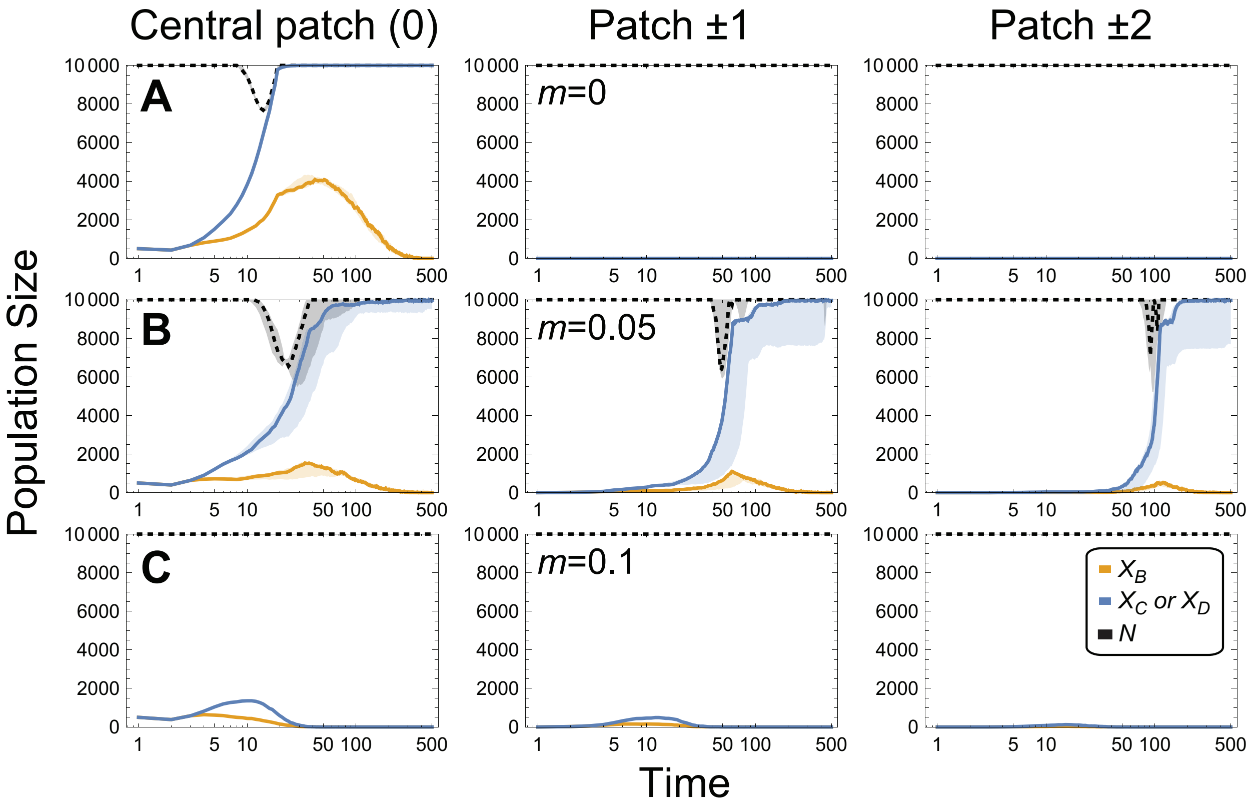

S7 Fig. Population modification with daisy quorum drive in finite populations. Black dashed curve is the total population size at time t, blue solid curve represents the number of individuals carrying the cargo allele C (equivalently D), and orange solid curve represents the number of individuals carrying the driver allele B. In this case, fertility is set to $F = 1.2$ and the payload to $s_p = 0.1$, so that subpopulations can persist even if the fitness-valley construct fixes. Each patch has a carrying capacity of $K = 10000$. Rows have a migration probability between adjacent patches of **(A)** $m = 0$, **(B)** $m = 0.05$, **(C)** $m = 0.1$ from top to bottom. Drive is released only in the central patch at frequency $f_0 = 0.05$ (left panels). The solid curves represent the median of 50 replicates, and the shaded regions the first and third quartiles.

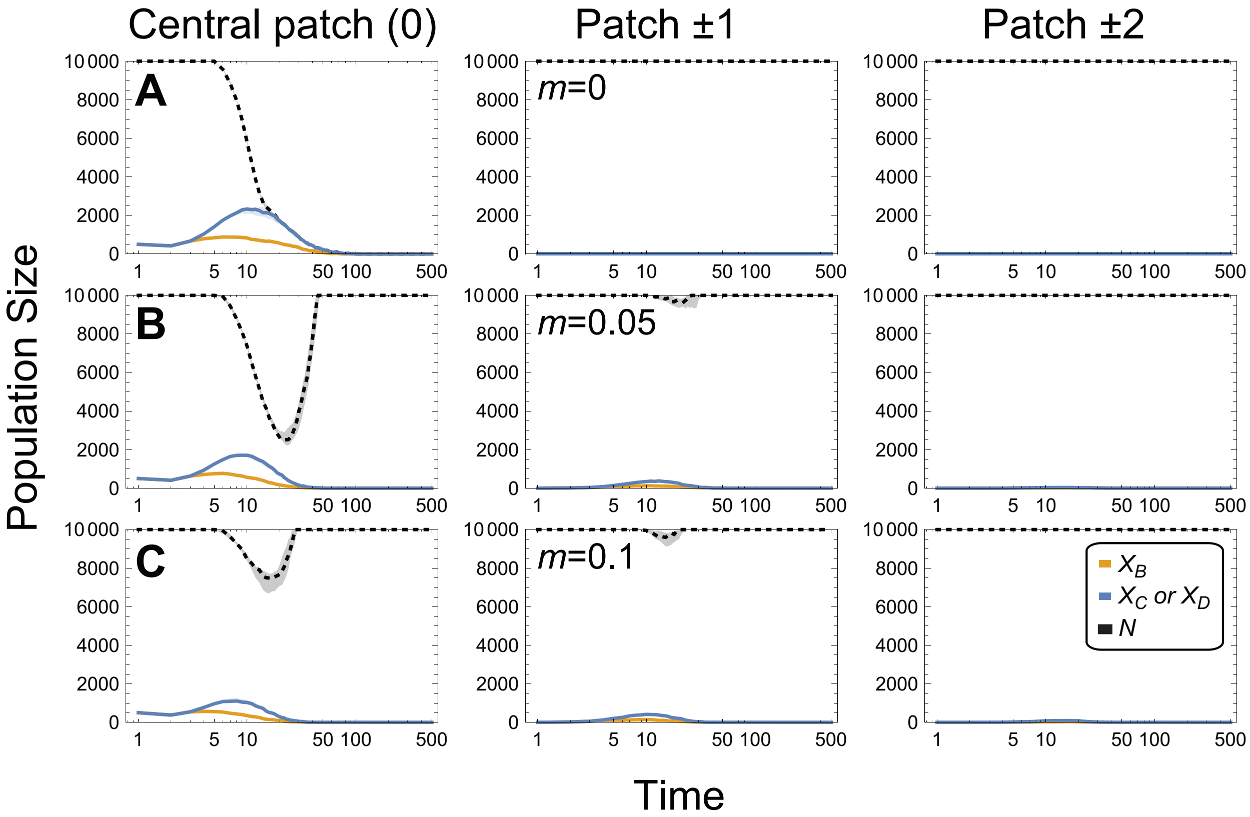

S8 Fig. Population suppression with daisy quorum drive in finite populations. Identical to S7 Fig but with fertility of $F = 1.05$, which is too low for a subpopulation to replace itself once the fitness-valley construct with a payload of $s_p = 0.1$ has fixed.
