# Supplementary material for: Modelling daisy quorum drive: A short-term bridge across engineered fitness valleys
Source: PLoS Genet. 2024 May 16;20(5):e1011262. doi: 10.1371/journal.pgen.1011262 (PMC11135765; doi:10.1371/journal.pgen.1011262)
Supplement: S3 Appendix — (PDF) [file pgen.1011262.s003.pdf]

Modelling daisy quorum drive: a short-term bridge across engineered fitness valleys (PLoS Genetics 2024)

Frederik JH de Haas, & Léna Kläy, Florence Débarre, Sarah P Otto*
* otto@zoology.ubc.ca

S3 Appendix. Numerical simulations in continuous space

S3.1 Relationship between discrete and continuous space

To model the dynamics of daisy quorum drive in a continuous environment along one or two spatial dimensions, we first calculate the change in frequency for each of the 16 gametes per small unit of time (Δt) within a spatial location, using the single population dynamics in S1 Appendix scaled to Δt. We then allow migration between adjacent locations by dividing space into small patches separated by a distance Δx. Finally, we shrink both the time interval and spatial interval to obtain a set of reaction-diffusion equations. In practice, these equations are solved numerically by discretizing time and space, using very small intervals to approximate the continuous dynamics.

For example, consider the dynamics for a population arrayed along one dimension, where population densities and migration rates are equal over space (the homogeneous case). From the discrete-time model without migration, let $p_i^{s,r}(t,x)$ equal the frequency of gamete i after one generation of selection and reproduction at generation t in a single location x, given the complex rules of daisy quorum drive as in S1 Appendix, with $\Delta p_i^{s,r}(t,x) = p_i^{s,r}(t,x) - p_i(t,x)$ being the change in frequency due to selection and reproduction within one generation (which will provide the reaction term of the reaction-diffusion equation). Subsequently allowing migration, the frequency of chromosome i in a non-boundary population at discrete spatial location x becomes:

$$p_i(t+1,x) = (1-m)\ p_i^{s,r}(t,x) + \frac{1}{2} m\ p_i^{s,r}(t,x-1) + \frac{1}{2}\ m\ p_i^{s,r}(t,x+1). \tag{8}$$

We then shrink the time interval to Δt and the spatial interval to Δx, taking m to be the rate of migration in these new units and $\Delta p_i^{s,r}(t,x)\Delta t$ to be the change in frequency due to selection and reproduction. The frequency of gametes of type i in patch x at time t then changes according to:

$$p_i(t+\Delta t,x) = (1-m)\ p_i(t,x) + \frac{1}{2}\ m\ p_i(t,x-\Delta x) + \frac{1}{2}\ m\ p_i(t,x+\Delta x) + \Delta p_i^{s,r}(t,x)\Delta t, \tag{9}$$

which assumes that the time interval is so short that only one event is likely to occur (either migration or selection and reproduction). Dividing by the time interval,

$$\frac{p_i(t+\Delta t,x) - p_i(t,x)}{\Delta t} = \frac{m(\Delta x)^2}{2\Delta t}\ \frac{p_i(t,x-\Delta x) - 2p_i(t,x) + p_i(t,x+\Delta x)}{(\Delta x)^2} + \Delta p_i^{sel}(t,x). \tag{10}$$

The first term clarifies the relationship between the migration rate and the diffusion rate:

$$\mathcal{D} = \frac{m(\Delta x)^2}{2\Delta t}.$$

Letting Δx and Δt approach zero in the continuous limit, we obtain a set of 16 partial differential equations for the 16 gametes:

$$\frac{\partial p_i}{\partial t} = \mathcal{D}\frac{\partial^2 p_i}{\partial x^2} + \Delta p_i^{s,r}(t,x), \tag{11}$$

as long as $m(\Delta x)^2/\Delta t$ tends to a finite constant value, meaning that the resulting diffusion rate $\mathcal{D}$ is constant for a fixed migration rate m.

When comparing discrete and continuous environments (i.e., varying the spatial distance between patches, Δx), we hold the diffusion rate constant per unit of distance, so that the migration rate between patches declines as the patches move further apart. We use Neumann boundary conditions as in section 2.4; that is, the boundary populations only give and receive migrants from the interior.

S3.2 Numerical simulations across a homogeneous landscape

We simulate the propagation of alleles A, B, C, and D over a one-dimensional continuous homogeneous domain with two different initial conditions (code archived on Zenodo doi:10.5281/zenodo.10904198). We consider the introduction of either fully modified individuals ($ABCD$, light blue) or partially modified individuals ($abCD$, dark blue), replacing all individuals in a small central region (locations 90-110 over a domain of length 200). The rest of the domain is full of wild-type individuals ($abcd$), and all the other genotypes are absent at $t = 0$. Numerically, the dynamics are approximated by taking small step sizes in both time (time steps of 0.1 generations) and space (using a default spatial step size of 0.1 unit, unless otherwise stated).

As time passes, the cargo wave (measuring either X_C or X_D, which remain equal in frequency) approaches a constant speed. The wave either continues to move outward with a positive asymptotic speed (top row of S9 Fig), or the wave collapses with a negative asymptotic speed (bottom row). Whether the asymptotic wave speed is negative or positive cannot be analytically predicted, as it is the result of four coupled reaction-diffusion equations (the four remaining after the daisy chain constructs have disappeared). The net result depends on whether the push outwards when the fitness-valley construct is at high frequency is stronger than the pull inwards when the construct is at low frequency. In our simulations, increasing the payload always decreases the asymptotic wave speed for a given toxin load.

Importantly, the presence of drive only transiently increases the speed of the wave; the asymptotic speed does not depend on the daisy chain (loci **A** and **B**), which stops driving the cargo as time passes (as seen in S9 Fig panels B and F, where the drive alleles are more restricted in space than the cargo and eventually disappear). Thus, as expected, the same asymptotic speed is reached in simulations started without a daisy chain and only alleles C and D (darker curves in right column of S9 Fig).

We investigated the hypothesis that the asymptotic behaviour transitions from a spreading wave (positive speed) to a collapsing wave (negative speed) when the unstable

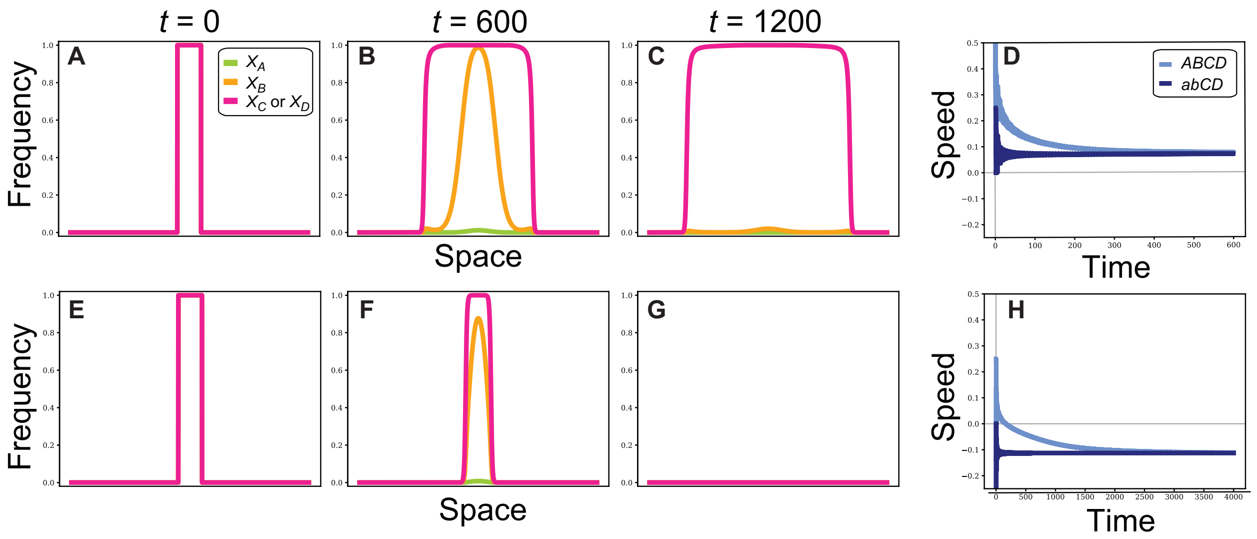

S9 Fig. Spread of introduced genotypes across one-dimensional continuous space that is homogeneous. The first three panels in each row represent allele frequencies in space at times **(A,E)** $t = 0$, **(B,F)** $t = 300$, and **(C,G)** $t = 600$. The right-most panels **(D,H)** show the speed of the X_C wave (or equivalently X_D wave) as a function of time. The rows illustrate simulations with a low payload (top row, $s_p = 0.1$) or a high payload (bottom row, $s_p = 0.5$). The simulations introduced fully modified individuals ($ABCD$) in the centre of the range, except that the last column compares the wave speed for this case (light blue) to the case where only the fitness-valley component ($abCD$) is introduced. Other parameters: $\delta = 0.9$ (drive rate), $s_d = 0.02$ (drive load), $s_t = 0.9$ (toxin load), $R = r = 0.5$ (recombination rate), $\mathcal{D} = 0.2$ (diffusion rate), and $L = 200$ (length of the spatial domain). Time and space were subdivided into ten steps each to approximate continuous time and space (i.e., using a spatial step size of 0.1).

equilibrium of the two-locus fitness-valley system crosses the midpoint where $p_C = p_c = p_D = p_d = 1/2$ (see Eq 3), as occurs in the one-locus fitness-valley system [44], but we found an imperfect match. For example, in S11 Fig with $s_t = 0.9$ and $r = 1/2$, the unstable equilibrium rises above the midpoint for $s_p > 0.44$, but the threshold payload needed for a negative asymptotic wave speed occurs over a broader range of $s_p > 0.3$. Thus, Eq 3 does not serve to determine the threshold for negative wave speeds, presumably because of the asymmetries in how the toxin load and payload interact (Table 1).

S3.3 Numerical simulations across a heterogeneous landscape

While daisy quorum drive cannot be stably maintained in a restricted region when space is homogeneous, spatial heterogeneity, such as variation in the viscosity of the environment to movement, can stabilize the system. Numerically, we explore this heterogeneity by discretizing space and time and allowing for different step sizes between adjacent patches.

First, consider an example where the landscape is easy to traverse in the centre of the range (spatial steps of size 1, with patch edges in grey close to one another) but challenging to traverse outside of this region (spatial steps of size 2). With $s_p = 0.1$, the critical step size for an expanding wave is around 1.7 (S11 Fig). Thus, in this case, the fitness-valley construct propagates outwards in a wave only until reaching the area where dispersal between sites becomes challenging (S10 Fig).

In the main text, we consider random spatial heterogeneity, where dispersal rates between

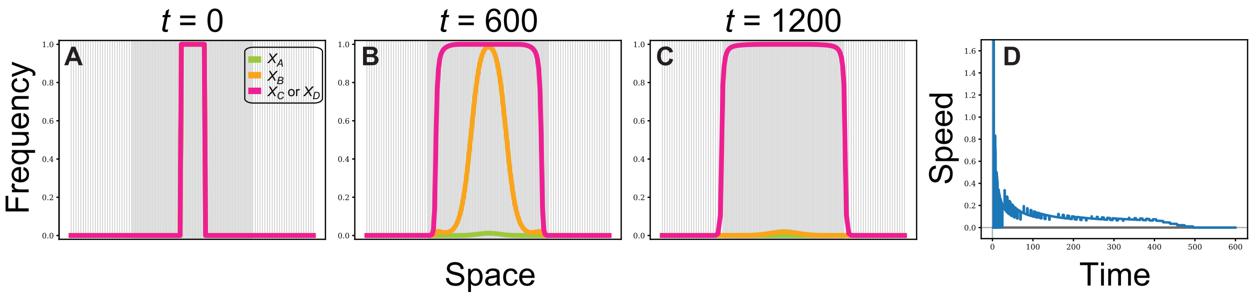

S10 Fig. Spread of introduced genotypes across one-dimensional continuous space that is heterogeneous. The first three panels represent alleles frequencies along a one-dimensional spatial axis at times **(A)** $t = 0$, **(B)** $t = 300$, and **(C)** $t = 600$. The last panel **(D)** describes the speed of the cargo wave, measuring the frequency of either C or D, i.e. the speed of the pink curve in the first three graphs. The domain is heterogeneous, with steps of size 1 (middle half) and steps of size 2 (outer half). Other parameters as in S9 Fig.

neighboring sites were determined by randomly placing 400 boundaries across a spatial domain of length 200. The step size separating adjacent regions then determines local dispersal rates, with higher dispersal between neighboring sites when the boundaries are closer. As seen in Fig 7 (bottom row), spatial heterogeneity can also stabilize waves that are collapsing once drive has been exhausted. In this case, regions of low dispersal prevent wildtype alleles from displacing the construct further, and the fitness-valley construct can be stably maintained in a localized region.

S3.4 Comparing asymptotic wave speeds between continuous and discrete space models

To better understand the differences between discrete patches and continuous space, we discretized space by concentrating individuals into fewer and fewer patches, with larger steps between them (S11 Fig). We held all else equal, including the population density and diffusion rate. As expected from the continuous-space model, with many patches and small steps between them (near zero on the x-axis), the wave reaches a steady asymptotic speed, either expanding or contracting depending on the payload. As the step size increases further, the wave speed decreases in absolute value, until a critical size is reached, above which the speed becomes zero.

In other words, in a discrete environment with large enough gaps between patches, the drive is confined and maintains itself around the area of introduction, whereas in continuous space it either expands (small payload cost s_p) or contracts (large payload cost s_p) for a given toxin load s_t. These results echo those of Barton [44], who determined the critical size of spatial steps analytically in the case of a single-locus fitness-valley model in one spatial dimension.

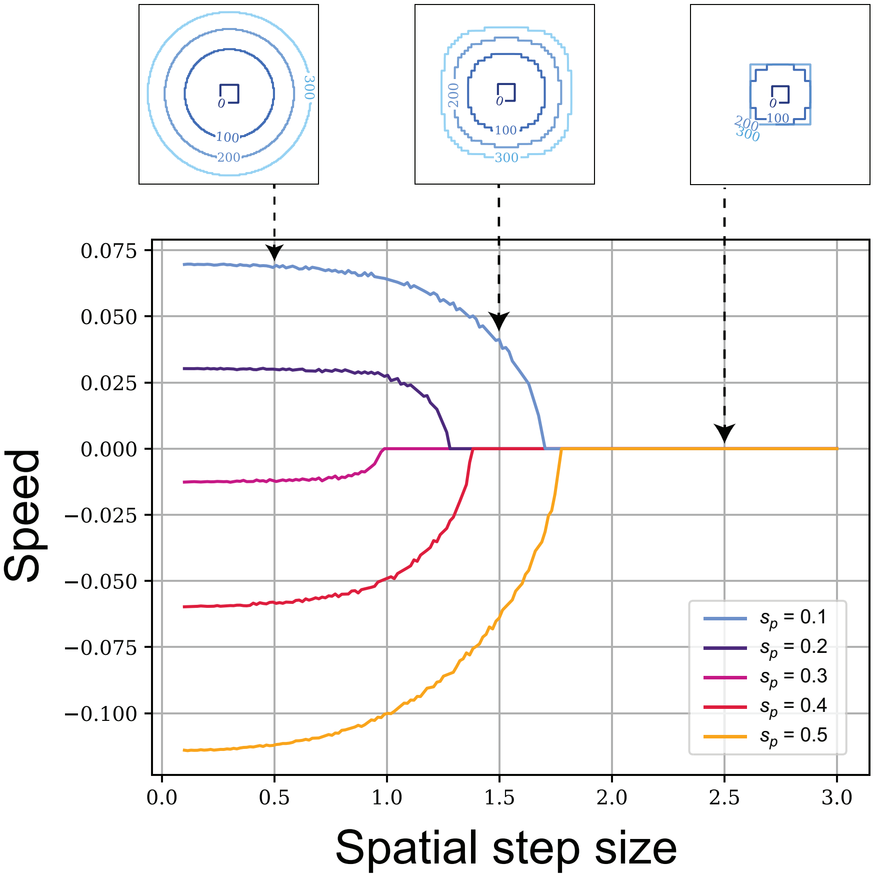

S11 Fig. The asymptotic wave speed for daisy quorum drive as a function of the spatial step size for different values of the payload cost s_p across a two-dimensional area. The three panels above the graph show the convex hull containing 80% of the population for either the C or D allele at $t = 0$ (dark blue contour), $t = 100$, $t = 200$ and $t = 300$ (light blue), with $s_p = 0.1$. Parameters are as follows: $R = r = 0.5$ (recombination rate), $s_d = 0.02$ (drive load), $s_t = 0.9$ (toxin load), $\delta = 0.9$ (drive rate), $T = 4000$ (final time), $L = 800$ (length of the spatial domain), $\mathcal{D} = 0.2$ (diffusion rate), with each generation split into ten time steps to mimic continuous time and the spatial domain split into a series of patches (from 8000^2 down to 267^2, across the two dimensions) at increasing distances apart (from 0.1 to 3 spatial units).
