# Supplementary material for: Modelling daisy quorum drive: A short-term bridge across engineered fitness valleys
Source: PLoS Genet. 2024 May 16;20(5):e1011262. doi: 10.1371/journal.pgen.1011262 (PMC11135765; doi:10.1371/journal.pgen.1011262)
Supplement: S1 Table — Table illustrates the gametes that come together to make a diploid individual (first two columns), their fitness (third column), frequency at birth (fourth column), and gametes produced (last four columns). (PDF) [file pgen.1011262.s015.pdf]

Gamete 1	Gamete 2	Fitness	Freq	Gametes produced			
				ab	aB	Ab	AB
ab	ab	1	X_{ab}^2	1			
ab	aB	$(1-s_d)$	$2X_{ab}X_{aB}$	$\frac{1}{2}$	$\frac{1}{2}$		
ab	Ab	$(1-s_d)$	$2X_{ab}X_{Ab}$	$\frac{1}{2}$		$\frac{1}{2}$	
ab	AB	$(1-s_d)^2$	$2X_{ab}X_{AB}$	$\frac{1}{2}(1-\delta)(1-r)$	$\frac{1}{2}(\delta+(1-\delta)r)$	$\frac{1}{2}(1-\delta)r$	$\frac{1}{2}(1-(1-\delta)r)$
aB	aB	$(1-s_d)^2$	X_{aB}^2		1		
aB	Ab	$(1-s_d)^2$	$2X_{aB}X_{Ab}$	$\frac{1}{2}(1-\delta)r$	$\frac{1}{2}(1-(1-\delta)r)$	$\frac{1}{2}(1-\delta)(1-r)$	$\frac{1}{2}(\delta+(1-\delta)r)$
aB	AB	$(1-s_d)^3$	$2X_{aB}X_{AB}$		$\frac{1}{2}$	$\frac{1}{2}$	
Ab	Ab	$(1-s_d)^2$	X_{Ab}^2			1	
Ab	AB	$(1-s_d)^3$	$2X_{Ab}X_{AB}$			$\frac{1}{2}(1-\delta)$	$\frac{1}{2}(1+\delta)$
AB	AB	$(1-s_d)^4$	X_{AB}^2				1

S1 Table. Mating table for loci A and B. Table illustrates the gametes that come together to make a diploid individual (first two columns), their fitness (third column), frequency at birth (fourth column), and gametes produced (last four columns).
