# Supplementary material for: Modelling daisy quorum drive: A short-term bridge across engineered fitness valleys
Source: PLoS Genet. 2024 May 16;20(5):e1011262. doi: 10.1371/journal.pgen.1011262 (PMC11135765; doi:10.1371/journal.pgen.1011262)
Supplement: S2 Table — Table illustrates the gametes that come together to make a diploid individual (first two columns), their fitness (third column), frequency at birth (fourth column), and gametes produced (last four columns). (PDF) [file pgen.1011262.s016.pdf]

Gamete 1	Gamete 2	Fitness	Freq	Gametes produced			
				cd	cD	Cd	CD
cd	cd	1	X_{cd}^2	1			
cd	cD	$(1-s_t)(1-s_p)$	$2X_{cd}X_{cD}$	$\frac{1}{2}$	$\frac{1}{2}$		
cd	Cd	$(1-s_t)(1-s_p)$	$2X_{cd}X_{Cd}$	$\frac{1}{2}$		$\frac{1}{2}$	
cd	CD	$(1-s_p)$	$2X_{cd}X_{CD}$	$\frac{1}{2}(1-r)$	$\frac{1}{2}r$	$\frac{1}{2}r$	$\frac{1}{2}(1-r)$
cD	cD	$(1-s_t)(1-s_p)$	X_{cD}^2		1		
cD	Cd	$(1-s_p)$	$2X_{cD}X_{Cd}$	$\frac{1}{2}r$	$\frac{1}{2}(1-r)$	$\frac{1}{2}(1-r)$	$\frac{1}{2}r$
cD	CD	$(1-s_p)$	$2X_{cD}X_{CD}$		$\frac{1}{2}$	$\frac{1}{2}$	
Cd	Cd	$(1-s_t)(1-s_p)$	X_{Cd}^2			1	
Cd	CD	$(1-s_p)$	$2X_{Cd}X_{CD}$			$\frac{1}{2}$	$\frac{1}{2}$
CD	CD	$(1-s_p)$	X_{CD}^2				1

S2 Table. Mating table for locus C and D. Table illustrates the gametes that come together to make a diploid individual (first two columns), their fitness (third column), frequency at birth (fourth column), and gametes produced (last four columns).
