# Supplementary material for: Modelling daisy quorum drive: A short-term bridge across engineered fitness valleys
Source: PLoS Genet. 2024 May 16;20(5):e1011262. doi: 10.1371/journal.pgen.1011262 (PMC11135765; doi:10.1371/journal.pgen.1011262)
Supplement: S3 Table — (PDF) [file pgen.1011262.s017.pdf]

	cd	cD	Cd	CD
cd	1	$(1-s_t)(1-s_p)^{1/4}$	$(1-s_t)(1-s_p)^{1/4}$	$(1-s_p)^{1/2}$
cD	$(1-s_t)(1-s_p)^{1/4}$	$(1-s_t)(1-s_p)^{1/2}$	$(1-s_p)^{1/2}$	$(1-s_p)^{3/4}$
Cd	$(1-s_t)(1-s_p)^{1/4}$	$(1-s_p)^{1/2}$	$(1-s_t)(1-s_p)^{1/2}$	$(1-s_p)^{3/4}$
CD	$(1-s_p)^{1/2}$	$(1-s_p)^{3/4}$	$(1-s_p)^{3/4}$	$1-s_p$

S3 Table. Fitnesses when expression of the payload (s_p) is multiplicative within and between loci.
