# Supplementary material for: Modelling daisy quorum drive: A short-term bridge across engineered fitness valleys
Source: PLoS Genet. 2024 May 16;20(5):e1011262. doi: 10.1371/journal.pgen.1011262 (PMC11135765; doi:10.1371/journal.pgen.1011262)
Supplement: S4 Table — (PDF) [file pgen.1011262.s018.pdf]

	cd	cD	Cd	CD
cd	1	$1 - s_t$	$1 - s_t$	1
cD	$1 - s_t$	$(1 - s_t)(1 - s_p)^{1/2}$	1	$(1 - s_p)^{1/2}$
Cd	$1 - s_t$	1	$(1 - s_t)(1 - s_p)^{1/2}$	$(1 - s_p)^{1/2}$
CD	1	$(1 - s_p)^{1/2}$	$(1 - s_p)^{1/2}$	$1 - s_p$

S4 Table. Fitnesses when expression of the payload (s_p) is recessive at locus C and at locus D, acting independently on each.
