# Supplementary material for: Modelling daisy quorum drive: A short-term bridge across engineered fitness valleys
Source: PLoS Genet. 2024 May 16;20(5):e1011262. doi: 10.1371/journal.pgen.1011262 (PMC11135765; doi:10.1371/journal.pgen.1011262)
Supplement: S5 Table — (PDF) [file pgen.1011262.s019.pdf]

	cd	cD	Cd	CD
cd	1	$(1-s_t)(1-s_p)$	$(1-s_t)(1-s_p)$	$(1-s_p)$
cD	$(1-s_t)(1-s_p)$	$(1-s_t)(1-s_p)$	$(1-s_p)$	$(1-s_t)(1-s_p)$
Cd	$(1-s_t)(1-s_p)$	$(1-s_p)$	$(1-s_t)(1-s_p)$	$(1-s_t)(1-s_p)$
CD	$(1-s_p)$	$(1-s_t)(1-s_p)$	$(1-s_t)(1-s_p)$	$(1-s_p)$

S5 Table. Fitnesses for a fitness valley created by swapping two haploinsufficient genes at alleles C and D, which also carry a dominant payload.
