# Supplementary material for: Modelling daisy quorum drive: A short-term bridge across engineered fitness valleys
Source: PLoS Genet. 2024 May 16;20(5):e1011262. doi: 10.1371/journal.pgen.1011262 (PMC11135765; doi:10.1371/journal.pgen.1011262)
Supplement: S6 Table — In those studies, the toxin load was set to st = 1. (PDF) [file pgen.1011262.s020.pdf]

	cd	cD	Cd	CD
cd	1	$(1 - s_t)$	$(1 - s_t)(1 - s_p)^{1/2}$	$(1 - s_p)^{1/2}$
cD	$(1 - s_t)$	$(1 - s_t)$	$(1 - s_p)^{1/2}$	$(1 - s_p)^{1/2}$
Cd	$(1 - s_t)(1 - s_p)^{1/2}$	$(1 - s_p)^{1/2}$	$(1 - s_t)(1 - s_p)$	$(1 - s_p)$
CD	$(1 - s_p)^{1/2}$	$(1 - s_p)^{1/2}$	$(1 - s_p)$	$(1 - s_p)$

S6 Table. Relative fitness values used by Dhole et al. [8] and Champer et al. [9]. In those studies, the toxin load was set to $s_t = 1$.
